# An endoscope with integrated transparent bioelectronics and theranostic nanoparticles for colon cancer treatment

**DOI:** 10.1038/ncomms10059

**Published:** 2015-11-30

**Authors:** Hyunjae Lee, Youngsik Lee, Changyeong Song, Hye Rim Cho, Roozbeh Ghaffari, Tae Kyu Choi, Kyung Hoon Kim, Young Bum Lee, Daishun Ling, Hyuk Lee, Su Jong Yu, Seung Hong Choi, Taeghwan Hyeon, Dae-Hyeong Kim

**Affiliations:** 1 Center for Nanoparticle Research, Institute for Basic Science (IBS), Seoul 151-742, Republic of Korea; 2 School of Chemical and Biological Engineering, Institute of Chemical Processes, Seoul National University, Seoul 151-742, Republic of Korea; 3 Department of Radiology, Seoul National University College of Medicine, Seoul 110-744, Republic of Korea; 4 MC10 Inc., 9 Camp Street, Cambridge, Massachusetts 02140, USA; 5 Institute of Pharmaceutics, College of Pharmaceutical Sciences, Zhejiang University, 866 Yuhangtang Road, Hangzhou 310058, China; 6 Division of Gastroenterology, Department of Medicine, Samsung Medical Center, Sungkyunkwan University School of Medicine, Seoul 135-710, Republic of Korea; 7 Department of Internal Medicine, Seoul National University College of Medicine, Seoul 110-744, Republic of Korea

## Abstract

The gastrointestinal tract is a challenging anatomical target for diagnostic and therapeutic procedures for bleeding, polyps and cancerous growths. Advanced endoscopes that combine imaging and therapies within the gastrointestinal tract provide an advantage over stand-alone diagnostic or therapeutic devices. However, current multimodal endoscopes lack the spatial resolution necessary to detect and treat small cancers and other abnormalities. Here we present a multifunctional endoscope-based interventional system that integrates transparent bioelectronics with theranostic nanoparticles, which are photoactivated within highly localized space near tumours or benign growths. These advanced electronics and nanoparticles collectively enable optical fluorescence-based mapping, electrical impedance and pH sensing, contact/temperature monitoring, radio frequency ablation and localized photo/chemotherapy, as the basis of a closed-loop solution for colon cancer treatment. *In vitro*, *ex vivo* and *in vivo* experiments highlight the utility of this technology for accurate detection, delineation and rapid targeted therapy of colon cancer or precancerous lesions.

## Abstract (web summary)

Current endoscopes are limited to detection or treatment of colon cancers and growths, or resolution is too low for clinical application. Here the authors present a multimodal endoscope with theranostic nanoparticles that integrates fluorescence-based mapping, electrical impedance, pH and temperature monitoring, RF ablation and localized phototherapy or chemotherapy.

## Graphical abstract

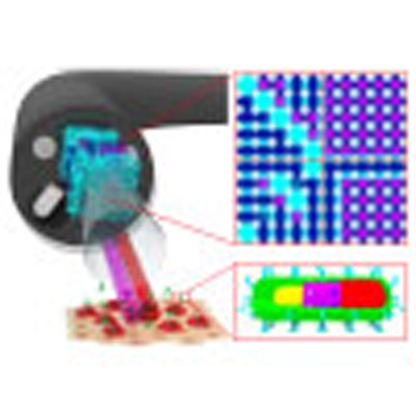

Conventional endoscopes consist of a flexible tube fitted with a camera, lens and light delivery system, providing both maneuverability and direct visualization of the gastrointestinal tract. More advanced endoscopes allow for enhanced flexibility and maneuvering within tightly spaced orifices, while offering both diagnostic and therapeutic capabilities including tissue biopsy and resection of tumours and polyps^1^. Despite the proven utility of current surgical endoscopes, onboard sensors coupled with treatments are unavailable because of the macro-scale size of conventional system, preventing diagnosis and therapy of micro-scale tumours. To understand the detailed physiological dynamics and treat cancerous tissues simultaneously *in vivo*, a new integrated system with targeted therapies and diagnostics is required^2^,^3^. Radio frequency (RF) ablation and localized photo-/chemotherapies with biocompatible theranostic nanoparticles serve as effective alternatives or a supplementation to surgical resection^4^,^5^. In both modes, intraprocedural mapping of diseased tissues using multimodal devices^6^,^7^,^8^,^9^ and controlled delivery/actuation of nanoparticles as imagers and probes^10^,^11^,^12^,^13^ for magnetic resonance, fluorescence, Raman scattering imaging provide precise information about the location and physicochemical properties of the targeted growth^14^,^15^,^16^,^17^. The small surface area of the endoscope tip, however, limits the number of useful sensing and therapeutic features that can be integrated on endoscopes using existing packaged electronics.

Here we demonstrate a multifunctional surgical endoscope system to diagnose and treat intestinal diseases, such as colon cancers. This ‘smart' endoscope system contains transparent bioelectronics, which provides impedance- and pH-based sensing, in combination with RF ablation therapy to facilitate the characterization and removal of colon cancers. Additional sensors for monitoring mechanical contacts and mapping temperatures provide accurate physiological sensing capabilities during cancer detection and ablation. The transparency of devices enables optimal integration of a number of multifunctional sensing and therapeutic components on the endoscope tip without blocking the line of sight of the camera or light. By loading transparent bioelectronics on the camera of the endoscope, the tissue observed through the camera in fluorescence mapping and/or phototherapies can be exactly matched with the characterized and/or ablated tissues by transparent devices. In addition to transparent bioelectronics, this system has custom-designed biocompatible theranostic nanoparticles (NPs) with phototherapeutic and chemotherapeutic agents, which can be delivered locally and activated with light. This multifunctional endoscopic system could be useful for the detection of flat or depressed neoplasms^18^ and for the treatment of patients with chronic inflammatory bowel diseases and with increased risks of developing malignancy due to undetected dysplastic lesions^19^. Synergistic effects between the transparent bioelectronics and theranostic NPs can enhance the tumour detection accuracy and provide treatment capabilities in response to the detection.

## Results

### Multifunctional endoscope system for colon cancer treatment

**Figure 1:**
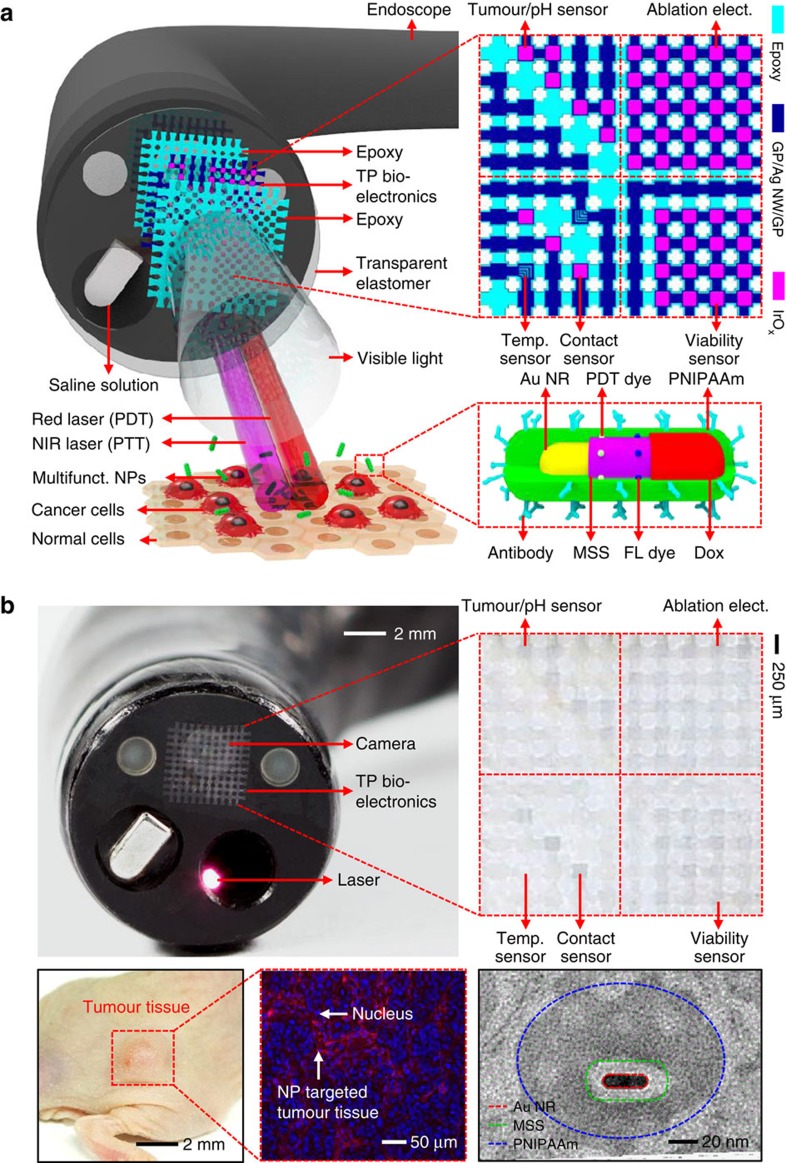
A multifunctional endoscope system. (**a**) Schematic illustrations of the design strategy and mode of use for the multifunctional endoscope system based on transparent bioelectronic devices and theranostic nanoparticles. (**b**) Images of the system corresponding to illustrations in **a**. Bottom frames show an optical camera image of NP-targeted HT-29 tumour grown in the mouse sub-dermis (left), a confocal microscope image of HT-29 tissues after NP targeting (middle) and a transmission electron microscope image of a designed theranostic NP (right). elect., electrode.

**Figure 2:**
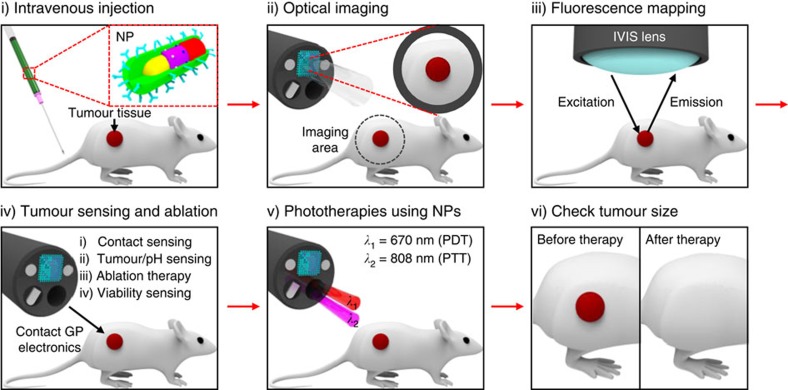
Tumour treatment procedures. Schematic illustrations of tumour treatment procedures with the multifunctional endoscope.

A representative clinical application of this smart endoscope system (Fig. 1a,b) involves the treatment of colon cancer as schematic illustrations described in Fig. 2. The treatment begins with the intravenous injection of NPs (Fig. 1a,b, bottom) that actively target colon cancer cells (HT-29) by specific antibody (cetuximab) that is conjugated on the surface of the NPs. Imaging of fluorescence dyes loaded on these NPs provides the optical information about the spatial distribution of cancer cells. The endoscope allows laser light to access suspicious sites exposed to NPs (Fig. 1a,b, top left). These regions are readily observed due to transparency of the integrated transparent bioelectronics on the endoscope camera, which have an overall transmittance of ∼80% in the visible range (Supplementary Fig. 1a,b). The transparent bioelectronics and associated sensors (Fig. 1a,b, top right) provide additional electrochemical analysis of the tumour distribution. The detailed design of electronics is shown in Supplementary Fig. 1c–e.

After a suspicious area of tissue is optically observed and potentially cancerous tissue is identified, these tissues are resected using forceps, followed by RF ablation using the transparent bioelectronics. Feedback modulations of this ablation therapy are based on the continuous monitoring of temperature, contact and cell/tissue viability. Photodynamic (PDT)-, photothermal (PTT)-, and chemotherapies induced by PDT dyes (chlorin e6; Ce6), gold nanorods (Au NRs) and chemo-drugs (doxorubicin; Dox) loaded in the mesoporous silica shell (MSS) can effectively destroy any residual cancer cells around the surgically treated area on activation with irradiated red or near-infrared (NIR) lasers. The thermosensitive poly(*N*-isopropylacrylamide) (PNIPAAm) shell prevents Dox from being released without NIR laser irradiations. Before procedures, the transparent bioelectronics are cleaned, sterilized^20^ and attached to the endoscope (Supplementary Fig. 2a,b). The sterilization procedure is performed using hot saturated steam in an autoclave (120 °C, 200 kPa, 15 min), which does not alter the performance of the electronics (Supplementary Fig. 2c). The repetitive application of normal and shear stress does not change the performance of electronics (Supplementary Fig. 2d). Note that the transparent bioelectronics can be mechanically bent and twisted without causing fractures (Supplementary Fig. 2e,f) during the installation, removal (Supplementary Fig. 2a,b and Supplementary Note 1) and wiring (Supplementary Fig. 3) due to the high degree of mechanical deformability achieved by neutral mechanical plane designs^21^,^22^, ultrathin structures^23^,^24^ and flexible properties of graphene (GP)^25^,^26^. The transparent bioelectronics also shows higher mechanical reliability than indium tin oxide, which is well-known material for transparent electrodes, but has brittle mechanical properties (Supplementary Fig. 2g). The simple attachment and detachment of the multifunctional transparent bioelectronic system provide the wide applicability to endoscopic devices. The multifunctional endoscopic system can be potentially used for the targeted ablation of unresectable multiple metastatic lesions^27^,^28^ or the natural orifice transluminal endoscopic surgery^29^ to detect and treat metastatic lesions as well as primary intraluminal lesions.

### Characterization of the graphene hybrid

**Figure 3:**
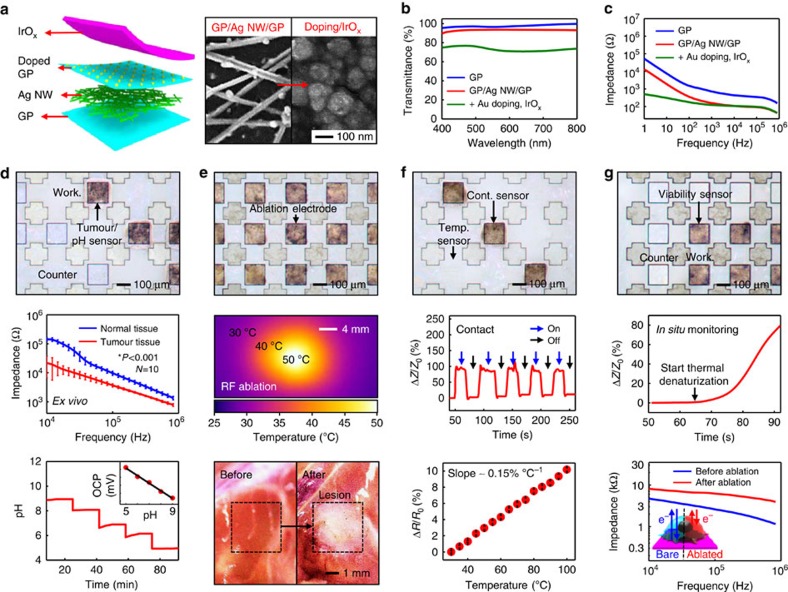
Transparent bioelectronics based on the graphene hybrid. (**a**) Schematic illustration of the graphene hybrid in the exploded view (left) and scanning electron microscope images before and after the IrO_x_ electrodeposition (right). (**b**) Optical transmittance measurement of the graphene hybrid. (**c**) Bode plots of the graphene hybrid. (**d**) Characterization of tumour and pH sensors (top: optical microscope image; middle: impedance measurement of tumour (HT-29) and normal tissues *ex vivo* (mouse number=10; *P<0.001, Student's *t*-test); bottom: pH monitoring in sequential additions of the acidic buffer solution). Working (Work.) and counter electrodes for electrochemical measurement are shown in the top frame. (**e**) Characterization of ablation electrodes (top: optical microscope image; middle: IR camera image during the RF ablation; bottom: optical camera images before and after RF ablations of mouse thigh tissues *ex vivo*). (**f**) Characterization of contact (Cont.) and temperature (Temp.) sensors (top: optical microscope image; middle: impedance measurements in on and off contacts; bottom: calibration curve of the temperature sensor). (**g**) Characterization of viability sensors (top: optical microscope image; middle: *in situ* impedance measurement during the thermal denaturization of mouse tissues; bottom: impedance measurement before and after the RF ablation of tumour tissues).

Figure 3a shows a schematic diagram and scanning electron microscope images of the graphene hybrid. Chemical vapour deposition-grown graphene^25^,^26^ is modified with Au chemical doping and iridium oxide (IrO_x_) deposition^9^. Au doping on graphene helps achieve uniform IrO_x_ plating (Supplementary Fig. 4 and Supplementary Note 2). Ag nanowires (Ag NWs) are then embedded to boost the electrical conductivity^30^. This hybrid structure provides good transparency (Fig. 3b). By selectively electroplating the IrO_x_ onto active sites, the device–tissue interface with low contact impedance is realized (Fig. 3c) with preserved high transparency. Additional characterization of graphene hybrid is included in Supplementary Fig. 5 and Supplementary Note 3. The stability of graphene hybrid immersed in biofluids at various temperatures (Supplementary Fig. 6; including hot steam for sterilization in Supplementary Fig. 2c) is confirmed through cyclic voltammetry tests.

### *In vitro* and *ex vivo* study of transparent bioelectronics

On the completion of microfabrication on a handle substrate (Supplementary Fig. 7), the transparent bioelectronics is transfer printed onto a preshaped PDMS segment (Supplementary Fig. 2a,b) and wired (Supplementary Fig. 3). Tumour/pH sensors, ablation electrodes and viability sensors are calibrated and characterized *ex vivo* using both resected HT-29 tissues and healthy tissues excised from the BALB/c nude mouse model. The tumour sensor is able to differentiate HT-29 tissues from normal tissues according to impedance differences (Fig. 3d, top and middle; Supplementary Note 4). Shift in pH levels around tumours due to the rapid cancer metabolization serves as another important marker used to detect tumours^31^. We monitor pH changes by measuring the open-circuit potential (OCP)^9^, since the surface zeta potential of graphene hybrid has the pH dependence (Fig. 3d, bottom; Supplementary Fig. 8a; and Supplementary Note 5). After the calibration, pH sensors show reliable and stable performances over different pH ranges, by different pH sensors, and in repeated multiple uses (Supplementary Fig. 8b–d). RF ablation studies using graphene hybrid electrodes are conducted *in vitro*, *ex vivo* and *in vivo* (Fig. 3e and Supplementary Fig. 9). The spatial (lateral and vertical) thermal distribution of RF ablation using graphene hybrid electrodes are compared with that using conventional commercial ablation electrodes (Boston Scientific Corporation; Model 5031T) on agar (*in vitro*) and BALB/c nude mouse model (*in vivo*), whose temperature distribution and lesion sizes are imaged with the infrared and optical camera, respectively (Supplementary Fig. 9). Both electrodes show similar results. To control lesion profiles effectively, conformal contact and temperature are constantly monitored during ablation (Fig. 3f). Finally, the viability sensor differentiates ablated tissues from non-ablated ones by measuring local impedance changes (Fig. 3g). Although the direct mapping of the three-dimensional thermal profile during RF ablation is challenging, it is clinically important. Previous reports provide a theoretical model for the prediction of the three-dimensional thermal profile during the ablation by using the two-dimensional surface temperature profile and the lesion size^6^.

### *In vitro* imaging and therapy with theranostic nanoparticles

**Figure 4:**
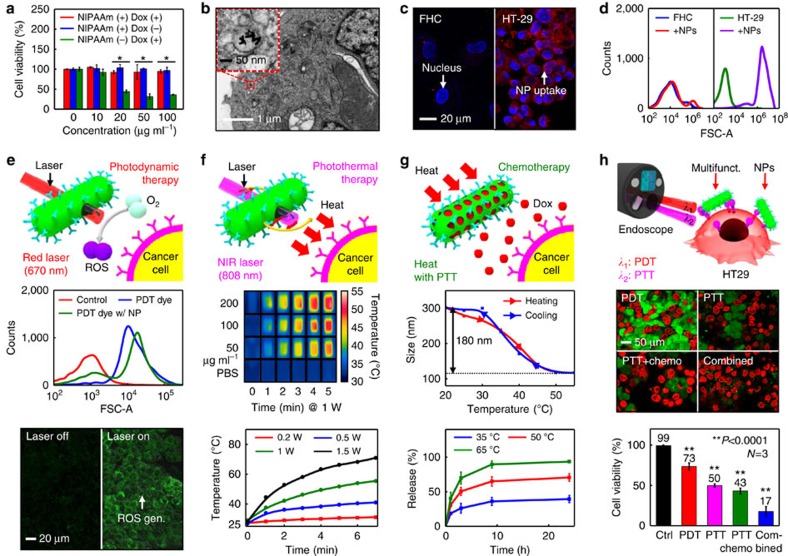
*In vitro* imaging and therapy using theranostic NPs. (**a**) Cell viability measurement of Dox-loaded NPs with (red) and without (green) the PNIPAAm encapsulation. Another control (Ctrl) group is Dox-unloaded NPs with PNIPAAm encapsulation (blue; *P<0.001, Student's *t*-test). (**b**) TEM image of the colon cancer (HT-29) cell after the targeted uptake of NPs. (**c**) Confocal microscope images (left: normal epithelial colon cell (FHC); right: colon cancer cell (HT-29) after the active targeting). Blue fluorescence areas show 4′,6-diamidino-2-phenylindole-dyed nucleus and red areas show rhodamine B-conjugated NPs. (**d**) Flow cytometry data of FHC and HT-29, which show the targeted uptake of NPs to HT-29 only. (**e**) Photodynamic therapy (top: schematic illustration; middle: quantitative comparison of cellular uptake of free PDT dyes and conjugated PDT dyes on NPs by flow cytometry; bottom: fluorescence images of ROS generation by using DHR123 dyes before and after continuous wave red laser radiation). (**f**) Photothermal therapy (top: schematic illustration; middle: IR camera images of NP suspensions of different concentrations under CW NIR laser radiations of various times; bottom: relationship between the radiation time and temperature for different laser powers at the constant NP concentration of 200 μg ml^−1^). (**g**) Chemotherapy (top: schematic illustration; middle: reversible change of the hydrodynamic diameter of NPs with respect to the temperature; bottom: thermally controlled drug release profiles). (**h**) Synergetic effect of multimodal phototherapies under pulsed laser irradiation (top: schematic illustration of the endoscope-guided phototherapy; middle: cell viability comparison of various treatments by using the calcein AM and propidium iodide assay; bottom: summary plot comparing the cell viability after various phototherapies) (**P<0.0001, Student's *t*-test). Multifunct., multifunctional.

Theranostic NPs, which are used in conjunction with the transparent bioelectronics, provide additional cancer diagnosis and targeted therapies (Fig. 1a,b, bottom). The detailed synthetic procedures and characterizations are described in Supplementary Fig. 10 and Supplementary Note 6. These NPs consist of Au NR core coated with MSS to create a photothermally active core-shell structure (Au NR@MSS)^15^,^32^, on which fluorescence dye (rhodamine B), PDT dye and Dox are loaded. PNIPAAm is polymerized on the surface of MSS to provide the effective encapsulation of Dox^10^,^33^. Cytotoxicity tests show that these drug-loaded NPs have minimal effect on the cell viability after the PNIPAAm encapsulation in comparison with control experiments (Fig. 4a). Cetuximab (Erbitux) antibody is conjugated on PNIPAAm to allow the active targeting (Figs 1b and 4b−d) of epidermal growth factor receptors that are overexpressed in colon cancer (HT-29) cells. The conjugation of the theranostic NPs with tumour-specific antibodies can distinguish the subtypes of tumour by fluorescence imaging^34^.

The cell transmission electron microscope images in Fig. 4b and Supplementary Fig. 11 show the targeted uptake of NPs by cancer cells, which is corroborated by fluorescence images in Fig. 4c and flow cytometry data in Fig. 4d. These theranostic NPs can treat cancer cells via reactive oxygen species (ROS) generation (Fig. 4e), photo-induced hyperthermia (Fig. 4f) and controlled drug release (Fig. 4g). The photoactivation of NPs is localized to laser-irradiated regions and controlled by modulating the laser intensity (Supplementary Figs 12–15). Direct control of the laser light, which is delivered through an optical fibre and guided with the endoscope (Fig. 1 and Supplementary Fig. 16), can overcome many issues related to the penetration depth of light^35^. Because colon cancers are normally located in superficial regions, our system is less affected by the penetration depth problem of light in comparison with other tumour cases. PDT dyes on NPs are more efficiently delivered to and taken up by cancer cells, compared with controls (cancer cells treated by free form of PDT dyes and non-treated ones), as shown in flow cytometry results (Fig. 4e, middle). When radiated by continuous wave red laser (wavelength 670 nm), PDT dyes generate ROS and cell viability is decreased (Fig. 4e, bottom; and Supplementary Figs 12b and 13)^36^. The temperature is photothermally modulated by changing particle concentration, continuous wave NIR laser intensity (wavelength 808 nm) and duration of irradiation, optimized for decreasing cancer cell viability (Fig. 4f and Supplementary Figs 12b and 14). Increasing temperature causes a change in the hydrodynamic diameter of NPs from ∼290 to ∼110 nm by the shrinkage of PNIPAAm layer, which in turn induces the release of Dox loaded in NPs (Fig. 4g, middle)^37^. The release temperature (>45 °C) is strategically designed to be higher than the body temperature (∼36.5 °C). PNIPAAm block copolymer suppresses drug release in the absence of laser radiation (Fig. 4g, bottom; and Supplementary Figs 12b and 15), thus minimizing side effects of Dox. Furthermore, viability tests of cancer cells after PDT, PTT, PTT/chemo- and combined therapies (using GaAs pulsed laser; wavelength 690 nm, power 30 mW for red laser and wavelength 808 nm, power 30 mW for NIR laser) confirm the synergistic effect of the combined treatment (Fig. 4h and Supplementary Fig. 12b).

### *In vivo* colon cancer treatment

**Figure 5:**
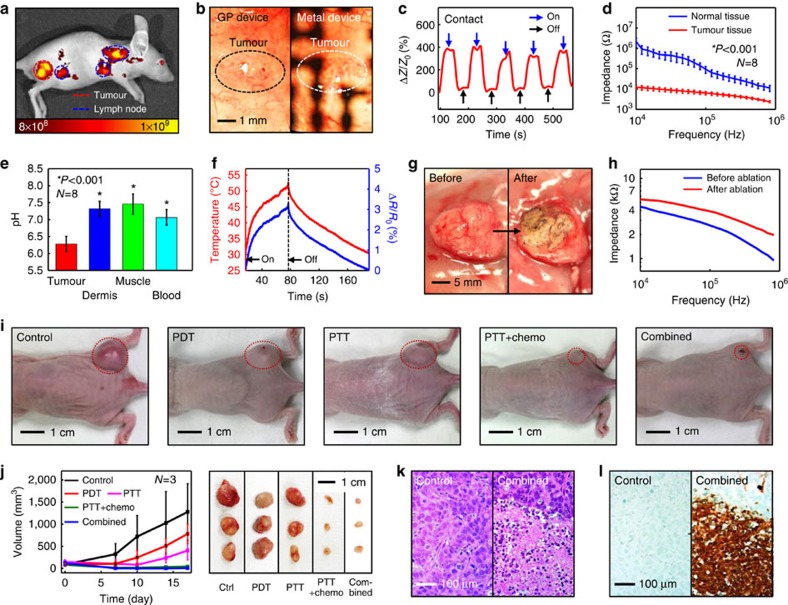
*In vivo* colon cancer treatment. (**a**) Merged fluorescence image of the colon cancer on the mouse sub-dermis 6 h after intravenous injection of NPs. (**b**) Images of the tumour, captured by the camera of the endoscope through electronic devices (left: through transparent bioelectronic devices; right: through control metal devices). (**c**) Contact sensing of tumours on the mouse sub-dermis. (**d**) Tumour detection using the subcutaneous colon cancer model in the BALB/c nude mouse (mouse number=8; *P<0.001, Student's *t*-test). (**e**) pH measurements of the tumour tissue, dermis tissue, muscle tissue and blood of the corresponding mouse model (mouse number=8; *P<0.001, Student's *t*-test). (**f**) *In situ* temperature measurement during the RF ablation therapy. (**g**) Images of tumour tissues before and after the RF ablation therapy. (**h**) Viability sensing before and after the ablation therapy of tumour tissues. (**i**) Images of the mouse model with HT-29 tumours after multimodal treatments (from left to right: control, PDT, PTT, PTT+chemo and combined therapy). Tumour volume changes are compared in each case and summarized in **j**. (**j**) Summary of tumour volume changes (left) and corresponding tumour images (right) of **i** (mouse number=3 for each). (**k**) Haematoxylin and eosin staining images of HT-29 tumour tissues of the control (Ctrl) group and the experimental group after combined therapy. (**l**) Terminal deoxynucleotidyl transferase dUTP nick end labelling assay of the control group and the experimental group after combined therapy.

The integrated system, transparent electronics on the endoscope and theranostic NPs actuated by guided lasers can be applied to *in vivo* models. The *in vivo* experimental set-up is shown in Supplementary Fig. 17. Endoscopic treatment of colon cancer (HT-29) grown on the sub-dermis surface of BALB/c nude mouse begins with the injection of NPs intravenously through the tail vein. Since colon cancer models are not available in large animals and endoscopes are too large for gastrointestinal tracts of small animals, we conduct *in vivo* studies using mouse subcutaneous colon cancer models. Side effects from Dox are minimized, however, since the release of Dox loaded on NPs is suppressed by the PNIPAAm encapsulation (Fig. 4a,g, bottom). Fluorescence image of resected organs and biodistribution analysis data show successful targeting (Supplementary Fig. 18a,b). Most NPs are cleared from the blood in 1 day due to the short circulation time (*t*_1/2_=20 min; Supplementary Fig. 18c). A large number of NPs accumulate in the tumour within 6 h of injection (Fig. 5a and Supplementary Fig. 18d) and active targeting using the antibody conjugation shows enhancement in the targeting efficiency of NPs (Supplementary Fig. 18e,f and Supplementary Note 7).

Fluorescence optical imaging of NP-targeted tumours is obtained *in vivo* by IVIS Lumina II (PerkinElmer) 6 h after NP injection, in which tumour-suspected and tumour-free areas are screened (Fig. 5a). The details are observed visually with the camera installed in the endoscope. Images of the tumour-grown surface captured by the endoscope camera reveal that the transparent bioelectronics do not disturb the visual observation, while the control standard metal device causes severe interferences (Fig. 5b). On identifying the cancerous cells, RF ablation therapy is employed to destroy the tumours. In this case, contact sensors are used to detect conformal contacts between electronics and tissues (Fig. 5c). In the contact mode during RF ablation, the visual observation of tumours is unavailable. Therefore, the impedance-based tumour and pH sensors are used to locate cancer tissues (Fig. 5d,e), according to the lower impedance and pH levels of tumour cells. The calibrated impedance and pH sensor successfully detects pH of target tissues *in vivo* under the contact mode (Fig. 5e). Monitoring temperature changes (Fig. 5f) provides additional guidance during RF ablation (Fig. 5g). Finally, tissue viabilities are measured to confirm the ablation therapy (Fig. 5h). We also conduct *ex vivo* studies using mouse colon cancer tissues attached on a porcine colon to show potential applicability of the current system to large animals (Supplementary Fig. 19). Although conducted *ex vivo*, these results validate operation of the device and demonstrate the potential for practical implementation in humans.

Together with physical treatments through the transparent bioelectronics, cancer cells are treated with theranostic NPs that are locally activated using continuous wave red and NIR lasers (Supplementary Fig. 16) to induce PDT (ROS), PTT (heat) and chemotherapy (Dox). The effectiveness of these multiple interventions is confirmed *in vivo* by tracking changes in tumour volume (HT-29) based on visual observations (Fig. 5i,j). Tumour volume increases in the control group (no therapy) after 2 weeks, whereas it decreases in the treated groups. Furthermore, when the tumour grown on the mouse model is either laser irradiated without injecting NPs or treated with chemo-drugs (Dox) only without targeting carriers (NPs), the tumour volume increases (Supplementary Fig. 20). The combined therapy group (PDT, PTT and chemotherapy all together) exhibits marked decrease in the tumour volume. Since the PTT+chemotherapy already has a good therapeutic effect and suppresses the tumour growth in our *in vivo* model, this makes little difference in tumour volume between PTT+chemo and combined therapies. However, the combined therapy shows its higher effectiveness than other therapies in the *in vitro* test (Fig. 4h). The haematoxylin and eosin (H&E) staining and terminal deoxynucleotidyl transferase dUTP nick end labelling (TUNEL) assay images of the tumour after treatments reveal irregular structures due to both apoptosis and necrosis of cancer cells (Fig. 5k,l and Supplementary Fig. 21). From 4′,6-diamidino-2-phenylindole and cleaved caspase-3 staining images, it seems that cell death might be caused by apoptosis (Supplementary Fig. 22)^38^,^39^. Although further large animal studies are required, the translation of theranostic nanoparticles for human patients can be pursued^13^.

## Discussion

Materials, designs and integration strategies for advanced, transparent bioelectronics and theranostic NPs onboard multifunctional endoscope systems have the potential to reduce the procedure time and improve the efficiency of minimally invasive surgical procedures for colon cancer treatment. These multifunctional endoscopic system for simultaneous *in vivo* histologic detection, delineation and rapid targeted treatment will be substantial for the reduction of missed lesions, whether they are intraluminal mucosal lesion or extraluminal metastatic lesion. Furthermore, efficient therapy can contribute to the excellent oncologic and economic yield for various gastrointestinal cancers or precancerous lesions in the future. These systems facilitate access to internal organs and provide a significant amount of diagnostic feedback during treatment routines, thus highlighting the utility of this technology in the translational medicine.

## Methods

### Fabrication and transfer of transparent bioelectronics

The microfabrication process (Supplementary Fig. 7) begins with the deposition of a sacrificial nickel layer (100 nm, via thermal evaporation) on a handle silicon wafer. Then a bottom epoxy layer is patterned using SU8-2. The graphene is synthesized on the Cu foil through a chemical vapour deposition process^26^. A Raman spectroscopy data of graphene is obtained using T64000 (Horiba, Japan) at National Center for Inter-University Research Facilities (NCIRF) (Supplementary Fig. 5a). The synthesized graphene is transferred to the target substrate by the graphene (GP) scooping method using poly(methyl methacrylate) A4 after the wet etching of the Cu foil. Next, the Ag NW solution (0.5 wt% in isopropanol) is spin coated at 2,000 r.p.m., followed by the annealing at 130 °C for 1 min. Ag NW/GP layers are patterned by a photolithographic process. Ag NWs on the active sites of the temperature sensors are selectively removed to enhance the temperature monitoring sensitivity (Supplementary Fig. 6b). Then, another GP layer is transferred onto Ag NW/GP layers. After patterning of the top graphene, the device is encapsulated by epoxy layer (SU8-2). External interconnection parts are also fabricated for power supply and data acquisition (Supplementary Fig. 3). These interconnections consist of a polyimide encapsulation layer and an Au/Cr metal layer, which is evaporated thermally and patterned via photolithography and etching processes. The fabricated transparent bioelectronics is transferred onto endoscope. In Fig. 1b and Supplementary Fig. 3, we use the Fujinon endoscope (ES-410WE, Fujinon; 13.0 mm of diameter, 760 mm of working channel length, flexible type) for imaging integrated devices on the endoscope. In Fig. 5b, we use the Olympus endoscope (CF Type H260AL, Olympus; 13.2 mm of diameter, 1,680 mm of working channel length, flexible type) for imaging the tumour by the camera of the endoscope through devices.

### Electroplating IrO_x_ for graphene hybrid

Before the electroplating of IrO_x_, GP/Ag NW/GP electrodes are immersed in a 20-mM AuCl_3_ solution for 10 min for doping, which enhances the uniformity of the IrO_x_ film deposition (Supplementary Fig. 4). The IrO_x_ solution for electroplating is prepared by dissolving 150 mg of iridium tetrachloride in 100 ml of ultrapure distilled water with 20 min of stirring. A 1-ml aliquot of aqueous 30% H_2_O_2_ is added and stirred for 10 min, and after which 500 mg of oxalic acid dihydrate is added and stirred for another 10 min. Finally, anhydrous potassium carbonate is used to adjust the solution pH to 10.5. The resulting solution is stored in room temperature for 1 week to stabilize iridium ions, resulting in a solution of the light-violet colour. Electrodeposition is performed by the three-electrode method using an electrochemical analyser. Chronopotentiometry is conducted at 0.7 V for 5 min across the graphene hybrid working electrode, the Pt wire counter electrode and the Ag/AgCl reference electrode in the IrO_x_ solution.

### Electrical stability of graphene hybrids in biofluids

To be suitable as sensors and actuators (for example, ablation electrodes) in a smart endoscope procedure, the electronics have to withstand temperature fluctuations during RF ablation and exposure to multiple electrochemical cycles in bio-fluidic environments. The graphene hybrid maintains a stable impedance value in the temperature range of 20–50 °C in PBS (Supplementary Fig. 6a). The change in the resistance of the interconnection is small (Supplementary Fig. 6b). The graphene hybrid maintains electrochemical stability after multiple cyclic voltammetry tests in PBS (Supplementary Fig. 6c). The stability of its impedance in fetal bovine serum (Life Technologies, 16000) is also confirmed over 6 h (Supplementary Fig. 6d), demonstrating stable electrochemical operation in bio-fluidic environments.

### *Ex vivo* and *in vivo* tumour sensing

*Ex vivo* and *in vivo* tumour sensing is based on the detection of different impedance values between resected normal tissues and tumours (HT-29) (Fig. 3d, middle and 5d). Tumours are grown on the thigh region of a mouse (BALB/c nude mouse). Normal tissues of the opposite thigh region are used as a control group. The resected tissues are positioned on the working and counter electrodes of tumour sensor (Fig. 3d, top), which are connected to an electrochemical analyser for impedance measurements (two-electrode method). *In vivo* impedance-based tumour sensing requires conformal contacts to target tissues for precise measurements. After incision of skin, the tumour sensor directly contacted to target tumour and normal tissues. The conformal contact is confirmed with integrated contact sensors.

### *In vitro* and *in vivo* pH sensing

The zeta potential of the IrO_x_ film surface is dependent on the pH of the solution and directly affects the OCP. The pH dependence of surface zeta potential is characterized using an electrokinetic analyser (Supplementary Fig. 8a). The pH sensor is connected to an electrochemical analyser and operated by two-electrode method using pH-sensitive graphene hybrid working electrode and Au-doped GP/Ag NW/GP counter electrode. The OCP is calibrated using standard buffer solutions (pH 5, 7 and 9 solution, Alfa Aesar; product #42417, 38172 and 42421) (inset of Fig. 3d, bottom). The reliability of pH sensors are tested with 10 pH sensors in repetitive uses using graphene hybrid working electrode, platinum counter electrode and Ag/AgCl reference electrode (Supplementary Fig. 8b–d). The measured OCP values are converted to pH values based on the calibration curve. During the *in vivo* experiment, pH sensor is directly contacted to the target tissues (tumour, dermis and muscle) after incision of skin and collection of blood from the mouse (Fig. 5e). For the pH measurement of blood, heparin is added to prevent coagulation of the collected blood (5 IU heparin per ml of blood).

### RF ablation and feedback monitoring

RF ablation is conducted using the experimental set-up shown in Supplementary Fig. 17, in which the transparent bioelectronics are connected to three different analysers. An electrochemical analyser is used to measure the impedance change of the contact sensor, the tumour detector and the viability sensor, which are all made from the same graphene hybrid but have different designs for each specific application. The RF ablation is conducted by connecting the ablation electrode to a RF generator and using the conventional RF ablation conditions (45–60 W). The on and off contact is monitored through impedance changes of the contact sensor. The temperature during the RF ablation is continuously monitored by measuring resistance changes of the temperature sensor by a digital multimeter, which is confirmed by a commercial infrared camera. Tissue viability is measured impedance change through pre- and post-RF ablation.

### Synthesis of multifunctional theranostic NPs

Multifunctional theranostic NPs are synthesized by multiple stepwise reactions and separation processes. The synthetic process consists of (i) Au NR synthesis, (ii) MSS synthesis, (iii) PDT and FL dye conjugation, (iv) PNIPAAm encapsulation and (v) antibody conjugation and Dox loading.

- Au NR synthesis: Au seed solution is made by injecting the NaBH_4_ solution (600 μl, 10 mM) to an aqueous seed solution containing HAuCl_4_·3H_2_O (250 μl, 10 mM) and cetyltrimethylammonium bromide (CTAB; 7.5 ml, 100 mM). The growth solution is made by adding HAuCl_4_·3H_2_O (1.7 ml, 10 mM) and AgNO_3_ (250 μl, 10 mM) to the CTAB solution (40 ml, 100 mM), into which L-ascorbic acid (270 μl, 100 mM) is injected. This Au seed is then converted to Au NR by injecting the additional seed solution (420 μl) into a growth solution, which is left to react for 3 h. The final product solution is centrifuged twice.
- MSS synthesis: the silica shell is grown on the surface of Au NR. Tetraethyl orthosilicate (30 μl) is injected to the Au NR solution (50 ml) under alkaline conditions (pH 10–11) and reacted with Au NRs for 4 h. Functionalization of the silica surface is achieved by injecting (3-aminopropyl) triethoxylsilane (10 μl) and 3-(methacryloxy) propyl triethoxysilane (10 μl), and the solution is stirred for 4 h. Then, the silica-coated Au NRs are centrifuged twice and dispersed in the ethanol. To create pores in the silica shell, HCl is added to the NP–ethanol suspension to adjust its pH to 1–2, which is refluxed to remove the CTAB templates. The resulting Au NR@MSS is centrifuged twice and dispersed in water.
- PDT and FL dye conjugation: Ce6 reacts with the equimolar amount of *N*-(3-dimethylaminopropyl)-*N*′-ethylcarbodiimide hydrochloride and *N*-hydroxysuccinimide (NHS). And the functionalized Ce6 is reacted with Au NR@MSS for 12 h. To conjugate FL dyes, rhodamine B isothiocyanate is mixed and reacted for 12 h. After the conjugation, Au NR@MSS is centrifuged and dispersed in water.
- PNIPAAm encapsulation: to produce a PNIPAAm shell, the Au NR@MSS solution (5 ml) is reacted with *N*-isopropylacrylamide (12 ml, 100 mM), acrylic acids (1.4 ml, 100 mM), *N*, *N*′-methylenebis(acrylamide) (1.2 ml, 100 mM), acrylate–PEG–NHS (20 mg) ans SDS (200 μl). The solution is bubbled with argon and heated to 70 °C to remove oxygen. After 30 min, potassium persulfate (1 ml, 20 mM) is injected to initiate the polymerization. The Au NR@MSS@PNIPAAm solution is then centrifuged twice to remove unreacted chemical regents.
- Antibody conjugation and Dox loading: the cetuximab (antibody) (2 ml, 5 mg ml^−1^) is added to the Au NR@MSS@PNIPAAm solution for the conjugation. The NHS-end group is reactive with the PEG-end group. This antibody-conjugated Au NR@MSS@PNIPAAm is then centrifuged and dispersed in PBS. Then, Dox (1 ml, 0.6 mg ml^−1^) solution is added to the NP solution (5 ml) and stirred for 1 day. Excess Dox is removed by centrifuging NPs.

### Characterization of theranostic NPs

The size changes of the synthesized NPs according to the temperature change (increase from 20 to 55 °C and decrease from 55 to 20 °C) are measured by the dynamic light scattering. The hydrodynamic diameter is measured for every 5 °C increment (Fig. 4g, middle). Temperature changes in the Au NR and PBS solution (concentration of 50, 100 and 200 μg ml^−1^) induced by the continuous wave NIR laser irradiation (1 W, 5 min) are measured by the infrared camera at 0, 1, 2, 3, 4 and 5 min (Fig. 4f, middle and bottom). Ultraviolet–visible spectrophotometry is used to calculate the amount of drug loaded on NPs by measuring the initial and supernatant Dox peak intensity values at 480 nm. Photoluminescence spectrophotometry is used to acquire the amount of released drug by measuring supernatant Dox peak intensity value at 580 nm (excitation wavelength of 480 nm) (Fig. 4g bottom). Florescence correlation spectroscopy is used to calculate the amount of conjugated FL dyes on NPs. The amount of PDT dyes conjugated on NPs is measured by using ultraviolet–visible spectrophotometer and ICP-AES (inductively coupled plasma atomic emission spectroscopy).

### Preparation of FHC and HT-29 for *in vitro* experiments

Human colon epithelial normal cells (FHC) were purchased from American Type Culture Collection (ATCC; catalogue number: CRL-1831) and human colon epithelial cancer cells (HT-29) were purchased from Korean Cell Line Bank (catalogue number: 30038). The culture medium for the FHC (ATCC, CRL-1831) is DMEM/F-12 (Life Technologies, 11320) with 10% bovine serum (Life Technologies, 16000), 1% penicillin streptomycin, 25 mM HEPES, 10 ng ml^−1^ cholera toxin, 5 μg ml^−1^ insulin, 5 μg ml^−1^ transferrin and 100 ng ml^−1^ hydrocortisone. The culture medium for the HT-29 (KCLB, 30038) is RPMI 1640 containing 10% bovine serum and 1% penicillin streptomycin. Both cell types are deposited on culture plates and incubated at 37 °C under the atmosphere of 5% CO_2_.

### Cytotoxicity and active targeting of NPs *in vitro*

The cytotoxicity of the NPs is measured by MTS assay, in which HT-29 cells are suspended in the culture medium with a concentration of 1 × 10^5^ cells per ml. A volume of 100 μl of this suspension is dispensed in each well of a 96-well plate. After the incubation for 2 days, Dox-loaded and -unloaded NPs with the PNIPAAm encapsulation as well as Dox-loaded NPs without the PNIPAAm encapsulation are injected to check the cytotoxicity. After 1 day, each cell is washed and 100 μl of the fresh cell culture medium is added along with 20 μl of the MTS solution (CellTiter 96 AQ_ueous_). After further incubation for 2 h, the light absorbance at 490 nm, which is proportional to the cell viability, is measured by a cell plate reader (Fig. 4a). To estimate the active targeting efficiency, NPs are injected into the FHC and HT-29 cell media. Cells are incubated for 4 h and washed twice to remove remaining NPs. Since NPs are conjugated with a red fluorescence dye (RITC), their active targeting efficiency can be characterized by the red fluorescence mapping (*λ*_ex_/*λ*_em_=550/580 nm) using the confocal laser scanning microscopy (CLSM). The cell nuclei are also dyed with 4′,6-diamidino-2-phenylindole to identify the location of individual cells (*λ*_ex_/*λ*_em_=340/488 nm; Fig. 4c). The uptake of NPs by FHC and HT-29 is measured by a flow cytometer (Fig. 4d), in which 10,000 cells are counted. The targeting efficiency of free Ce6 and Ce6-conjugated NPs is also compared (Fig. 4e, middle). ROS generation after irradiation of continuous wave red laser is measured with ROS-sensing dye (DHR123, *λ*_ex_/*λ*_em_=488/520 nm) using CLSM (Fig. 4e, bottom).

### *In vitro* phototherapy experiments using theranostic NPs

The therapeutic effect of NPs is estimated by treating HT-29 cells with NPs, which are not conjugated with RITC, incubating cells for 4 h and then washing them twice to remove remaining NPs. In the case of PTT, NPs without the Dox loading are used. For the chemotherapy, Dox-loaded NPs are used. A pulsed laser (wavelength 690 nm, power 0–40 mW for PDT; wavelength 808 nm, power 0–40 mW for PTT) is used and irradiated for 10 min. A volume of 10 μl of calcein AM (1 mg ml^−1^) and propidium iodide (1 mg ml^−1^) are added afterwards. CLSM is used to measure the cell viability based on the excitation/emission wavelengths of calcein AM (*λ*_ex_/*λ*_em_=488/520 nm) and propidium iodide (*λ*_ex_/*λ*_em_=550/620 nm).

### Animal experiment and animal model of HT-29 cancer

All procedures are approved by the Institutional Animal Care and Use Committee (IACUC) of the Biomedical Research Institute of Seoul National University Hospital, and all experimental procedures are performed according to the IACUC guidelines. HT-29 cancer cells (1 × 10^6^) in 50 μl of serum-free media are mixed with an equivalent volume of Matrigel (BD Biosciences). The mixture is subcutaneously injected into the flank of female BALB/c nude mouse (aged 6–8 weeks). The maximum weight of tumours did not exceed 10% of total body weight in accordance with the IACUC guidelines. Experiments were performed with tumour sizes of 100±30 mm^3^ (2 weeks after the tumour implantation). After phototherapy mice were divided into two groups. One group was used to monitor the efficacy of treatment. The size of tumours was checked every 3 days and mice were killed 20 days after treatment. Mice in the other group were used for immunohistochemistry and killed 7 days after treatment.

### *In vivo* active targeting and phototherapies of NPs

NPs are intravenously injected into the BALB/c nude mouse model with colon cancer, through the tail vein. The estimation of *in vivo* toxicity is based on histology analysis of the organs (Supplementary Fig. 23). Individual organs show no inflammations after NPs targeting. The tumour-targeting efficiency of NPs is characterized through pharmacokinetics, biodistribution and fluorescence studies. Blood samples are collected at 10 min, 30 min, 2 h, 6 h and 24 h after the NP injection. After 24 h, the mice are killed and the organs are collected for quantitative measurement of NP distribution. The collected blood and organ samples are dissolved by the acid solution and the Au concentration is measured by ICP-MS (Supplementary Fig. 18b,c). The fluorescent images of the whole body and the extracted organs are obtained using IVIS Lumina II (PerkinElmer) (Fig. 5a and Supplementary Fig. 18a,d,e). *In vivo* photo- and chemotherapies using NPs are performed with the radiation of red and NIR continuous wave lasers. The tumour region is irradiated by 670-nm red laser (500 mW for 6 min) for PDT or 808-nm NIR laser (1.5 W for 6 min) for PTT. After treatments, tumour sizes are measured by caliper for 2 weeks (tumour volume=*W*^2^ × *L*/2, *W*=width, *L*=length) (Fig. 5j and Supplementary Fig. 20).

## Additional information

**How to cite this article:** Lee, H. *et al*. An endoscope with integrated transparent bioelectronics and theranostic nanoparticles for colon cancer treatment. *Nat. Commun.* 6:10059 doi: 10.1038/ncomms10059 (2015).

## Supplementary Material

Supplementary Information Supplementary Figures 1-23, Supplementary Notes 1-7 and Supplementary References.

## References

[b1] Balog J. . Intraoperative tissue identification using rapid evaporative ionization mass spectrometry. Sci. Transl. Med. 5, 194ra93 (2013). 10.1126/scitranslmed.3005623 23863833

[b2] Tennant D. A., Duran R. V. & Gottlieb E. Targeting metabolic transformation for cancer therapy. Nat. Rev. Cancer 10, 267–277 (2010). 20300106 10.1038/nrc2817

[b3] Vanneman M. & Dranoff G. Combining immunotherapy and targeted therapies in cancer treatment. Nat. Rev. Cancer 12, 237–251 (2012). 22437869 10.1038/nrc3237 PMC3967236

[b4] Chu K. F. & Dupuy D. E. Thermal ablation of tumours: biological mechanisms and advances in therapy. Nat. Rev. Cancer 14, 199–208 (2014). 24561446 10.1038/nrc3672

[b5] Petros R. A. & DeSimone J. M. Strategies in the design of nanoparticles for therapeutic applications. Nat. Rev. Drug Discov. 9, 615–627 (2010). 20616808 10.1038/nrd2591

[b6] Kim D.-H. . Materials for multifunctional balloon catheters with capabilities in cardiac electrophysiological mapping and ablation therapy. Nat. Mater. 10, 316–323 (2011). 21378969 10.1038/nmat2971 PMC3132573

[b7] Kim D.-H. . Electronic sensor and actuator webs for large-area complex geometry cardiac mapping and therapy. Proc. Natl Acad. Sci. USA 49, 19910–19915 (2012). 23150574 10.1073/pnas.1205923109 PMC3523871

[b8] Xu L. . 3D multifunctional integumentary membranes for spatiotemporal cardiac measurements and stimulation across the entire epicardium. Nat. Commun. 5, 3329 (2014). 24569383 10.1038/ncomms4329 PMC4521772

[b9] Chung H. J. . Stretchable, multiplexed pH sensors with demonstrations on rabbit and human hearts undergoing ischemia. Adv. Healthc. Mater. 3, 59–68 (2014). 23868871 10.1002/adhm.201300124 PMC3969880

[b10] Zhang Z. . Near infrared laser-induced targeted cancer therapy using thermoresponsive polymer encapsulated gold nanorods. J. Am. Chem. Soc. 136, 7317–7326 (2014). 24773323 10.1021/ja412735p

[b11] Kim J., Piao Y. & Hyeon T. Multifunctional nanostructured materials for multimodal imaging, and simultaneous imaging and therapy. Chem. Soc. Rev. 38, 372–390 (2009). 19169455 10.1039/b709883a

[b12] Burns A., Ow H. & Wiesner U. Fluorescent core–shell silica nanoparticles: towards “Lab on a Particle” architectures for nanobiotechnology. Chem. Soc. Rev. 35, 1028–1042 (2006). 17057833 10.1039/b600562b

[b13] Phillips E. . Clinical translation of an ultrasmall inorganic optical-PET imaging nanoparticle probe. Sci. Transl. Med. 6, 260ra149 (2014). 10.1126/scitranslmed.3009524 PMC4426391 25355699

[b14] Lee J.-H. . Artificially engineered magnetic nanoparticles for ultra-sensitive molecular imaging. Nat. Med. 13, 95–99 (2007). 17187073 10.1038/nm1467

[b15] Vivero-Escoto J. L. . Silica-based nanoprobes for biomedical imaging and theranostic applications. Chem. Soc. Rev. 41, 2673–2685 (2012). 22234515 10.1039/c2cs15229k PMC3777230

[b16] Zhang K., Hao L., Hurst S. J. & Mirkin C. A. Antibody-linked spherical nucleic acids for cellular targeting. J. Am. Chem. Soc. 134, 16488–16491 (2012). 23020598 10.1021/ja306854d PMC3501255

[b17] Garai E. . A real-time clinical endoscopic system for intraluminal, multiplexed imaging of surface-enhanced Raman scattering nanoparticles. PLoS ONE 10, e0123185 (2015). 25923788 10.1371/journal.pone.0123185 PMC4414592

[b18] Kudo S. . Colonoscopic diagnosis and management of nonpolypoid early colorectal cancer. World J. Surg. 24, 1081–1090 (2000). 11036286 10.1007/s002680010154

[b19] Mayer R. . Colorectal cancer in inflammatory bowel disease. Dis. Colon Rectum 42, 343–347 (1999). 10223754 10.1007/BF02236351

[b20] Kuribara K. . Organic transistors with high thermal stability for medical applications. Nat. Commun. 3, 723 (2012). 22395614 10.1038/ncomms1721

[b21] Kim D.-H. . Stretchable and foldable silicon integrated circuits. Science 320, 507–511 (2008). 18369106 10.1126/science.1154367

[b22] Kim D.-H. . Epidermal electronics. Science 333, 838–843 (2011). 21836009 10.1126/science.1206157

[b23] Kaltenbrunner M. . An ultra-lightweight design for imperceptible plastic electronics. Nature 499, 458–463 (2013). 23887430 10.1038/nature12314

[b24] Sekitani T., Zschieschang U., Klauk H. & Someya T. Flexible organic transistors and circuits with extreme bending stability. Nat. Mater. 9, 1015–2022 (2010). 21057499 10.1038/nmat2896

[b25] Kim K. S. . Large-scale pattern growth of graphene films for stretchable transparent electrodes. Nature 457, 706–710 (2009). 19145232 10.1038/nature07719

[b26] Bae S. . Roll-to-roll production of 30-inch graphene films for transparent electrodes. Nat. Nanotechnol. 5, 574–578 (2010). 20562870 10.1038/nnano.2010.132

[b27] Kikuchi S. . Biological ablation of sentinel lymph node metastasis in submucosally invaded early gastrointestinal cancer. Mol. Ther. 20, 522–532 (2015). 10.1038/mt.2014.244 PMC4351467 25523761

[b28] Clark M. E. & Smith R. R. Liver-directed therapies in metastatic colorectal cancer. J. Gastrointest. Oncol. 5, 374–387 (2014). 25276410 10.3978/j.issn.2078-6891.2014.064 PMC4173041

[b29] Daher R., Choulilard E. & Panis Y. New trends in colorectal surgery: single port and natural orifice techniques. World J. Gastroenterol. 20, 18104–18120 (2014). 25561780 10.3748/wjg.v20.i48.18104 PMC4277950

[b30] Lee M.-S. . High-performance, transparent, and stretchable electrodes using graphene−metal nanowire hybrid structures. Nano Lett. 13, 2814–2821 (2013). 23701320 10.1021/nl401070p

[b31] Gallagher F. A. . Magnetic resonance imaging of pH in vivo using hyperpolarized ^13^C-labelled bicarbonate. Nature 453, 940–943 (2008). 18509335 10.1038/nature07017

[b32] Jana N. R., Gearheart L. & Murphy C. J. Wet chemical synthesis of high aspect ratio cylindrical gold nanorods. J. Phys. Chem. B. 105, 4065–4067 (2001).

[b33] Singh N. . Bioresponsive mesoporous silica nanoparticles for triggered drug release. J. Am. Chem. Soc. 133, 19582–19585 (2011). 21981330 10.1021/ja206998x PMC3295203

[b34] Adams G. P. & Weiner L. M. Monoclonal antibody therapy of cancer. Nat. Biotechnol. 23, 1147–1157 (2005). 16151408 10.1038/nbt1137

[b35] Kim S. . Near-infrared fluorescent type II quantum dots for sentinel lymph node mapping. Nat. Biotechnol. 22, 93–97 (2003). 14661026 10.1038/nbt920 PMC2346610

[b36] Ling D. . Multifunctional tumor pH-sensitive self-assembled nanoparticles for bimodal imaging and treatment of resistant heterogeneous tumors. J. Am. Chem. Soc. 136, 5647–5655 (2014). 24689550 10.1021/ja4108287

[b37] Yavuz M. S. . Gold nanocages covered by smart polymers for controlled release with near-infrared light. Nat. Mater. 8, 935–939 (2009). 19881498 10.1038/nmat2564 PMC2787748

[b38] Liu H. . Multifunctional gold nanoshells on silica nanorattles: a platform for the combination of photothermal therapy and chemotherapy with low systemic toxicity. Angew. Chem. Int. Ed. 50, 891–895 (2011). 10.1002/anie.201002820 21246685

[b39] Idris N. M. . In vivo photodynamic therapy using upconversion nanoparticles as remote-controlled nanotransducers. Nat. Med. 18, 1580–1586 (2012). 22983397 10.1038/nm.2933

