# Supplementary material for: An endoscope with integrated transparent bioelectronics and theranostic nanoparticles for colon cancer treatment
Source: Nat Commun. 2015 Nov 30;6:10059. doi: 10.1038/ncomms10059 (PMC4674684; doi:10.1038/ncomms10059)
Supplement: Supplementary Information — Supplementary Figures 1-23, Supplementary Notes 1-7 and Supplementary References. [file ncomms10059-s1.pdf]

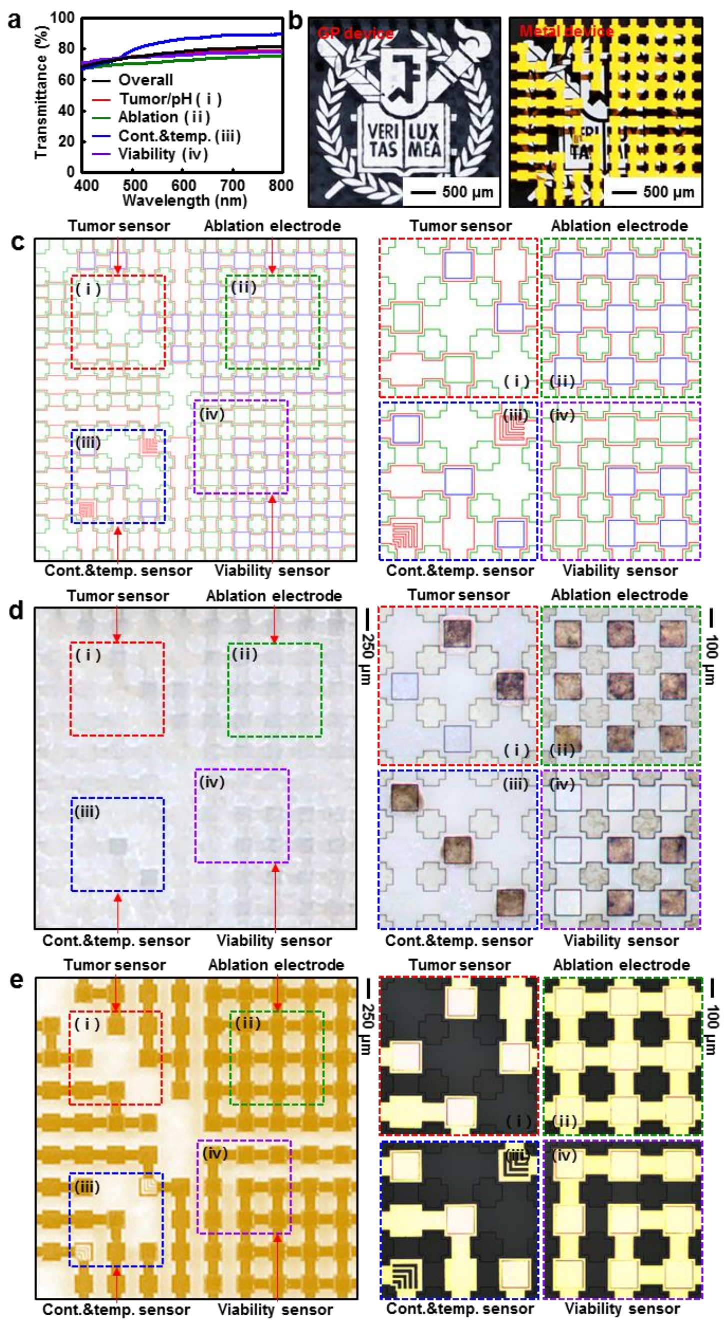

Supplementary Figure 1 | Transparency and detailed designs of transparent bio-electronics. (a) Transparency of each device region (see Supplementary Fig. 1c,d for (i), (ii), (iii), (iv)) in transparent bio-electronic devices. **(b)** Comparison of the transparency between transparent bio-electronic devices and gold-based ones (control). The university logo can be clearly seen through the transparent bio-electronic electronics. **(c)** CAD (computer aided design) of transparent bio-electronic devices. Right frames show the magnified view of dotted boxes ((i), (ii), (iii), (iv)). **(d)** Images of transparent bio-electronic devices corresponding to CAD in Supplementary Fig. 1c. **(e)** Images of gold-based devices corresponding to CAD of Supplementary Fig. 1c (control sample that clearly shows the pattern of Supplementary Fig. 1d).

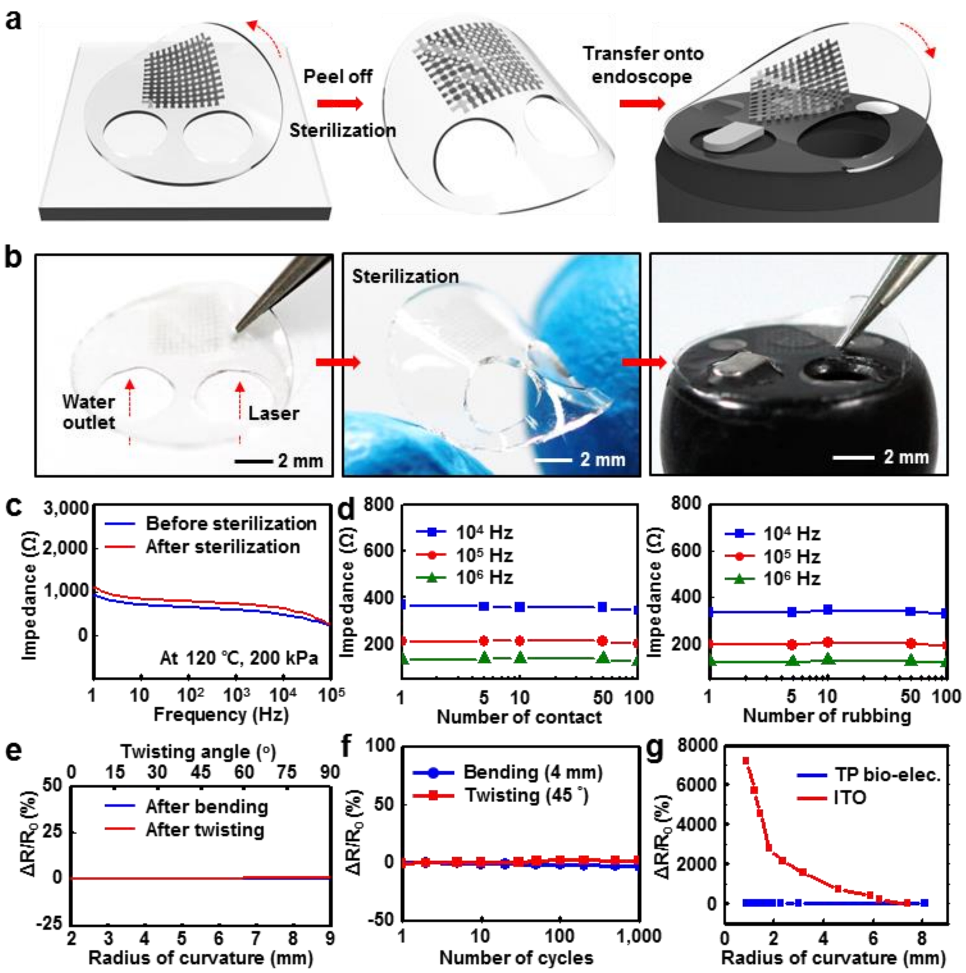

Supplementary Figure 2 | Integration process of the transparent bio-electronics on the endoscope and related characterizations. (a) Schematic illustration of the integration process of transparent bio-electronic devices on the endoscope. **(b)** Images of the integration process of transparent bio-electronic devices on the endoscope. **(c)** Impedance measurements before and after the sterilization in the autoclave (120 ^{o}C, 200 kPa, 15 min). **(d)** Impedance measurements in repetitive on/off-contact and rubbing tests on the pig intestine *ex-vivo*. **(e)** The mechanical stability of the transparent bio-electronics is shown by measuring percentage resistance changes of transparent bio-electronics after bending (blue line) and twisting (red line) deformations at the various radius of curvature. **(f)** The mechanical endurance of the transparent bio-electronics is shown by measuring percentage resistance changes of transparent bio-electronic devices in repetitive cycles. **(g)** Comparison of mechanical reliability between transparent bio-electronics and ITO electrode is shown by measuring percentage resistance changes under applied strains.

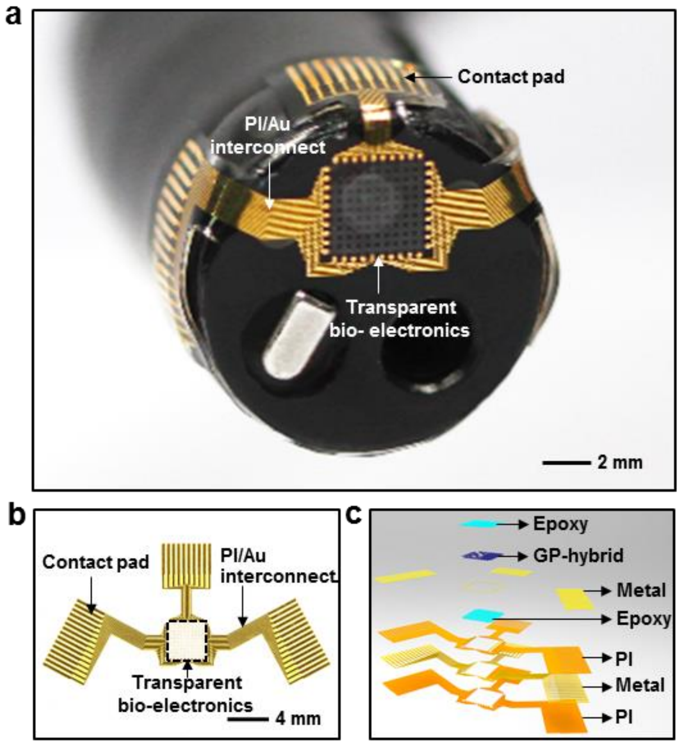

Supplementary Figure 3 | Transparent bio-electronic system installed on the endoscope with the external interconnection. (a) Transparent bio-electronics installed on the endoscope. Flexible interconnections are separately fabricated and installed. **(b)** Image of flexible interconnections for the transparent bio-electronics. **(c)** Schematic illustration of flexible interconnections (exploded view) for the transparent bio-electronics.

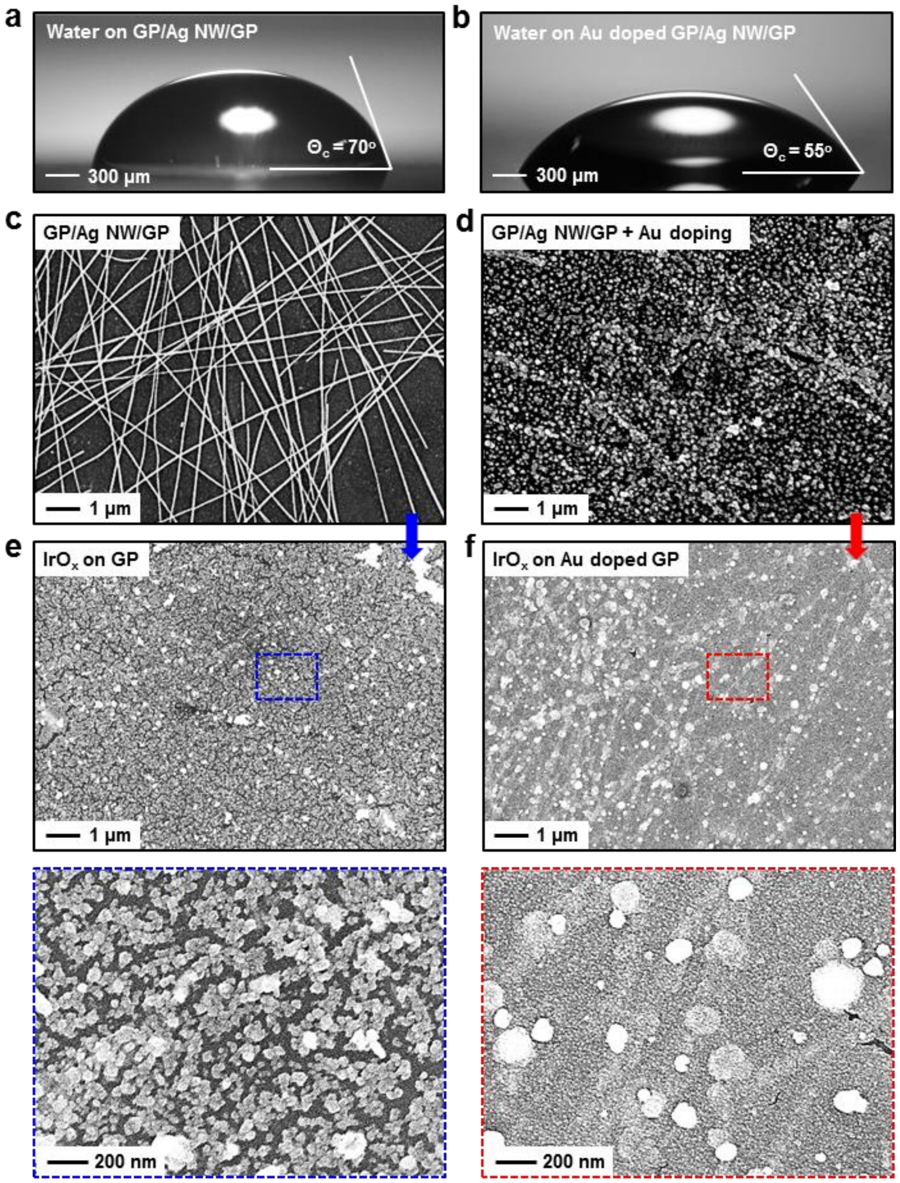

Supplementary Figure 4 | Comparison of the IrO_x film quality without (left column) and with (right column) Au doping. (a) Water contact angle of GP/Ag NW/GP. **(b)** Water contact angle of Au doped GP/Ag NW/GP. **(c)** SEM image of GP/Ag NW/GP. **(d)** SEM image of Au doped GP/Ag NW/GP. **(e)** SEM images of the IrO_x film electroplated on GP/Ag NW/GP. **(f)** SEM images of the IrO_x flim electroplated on Au doped GP/Ag NW/GP.

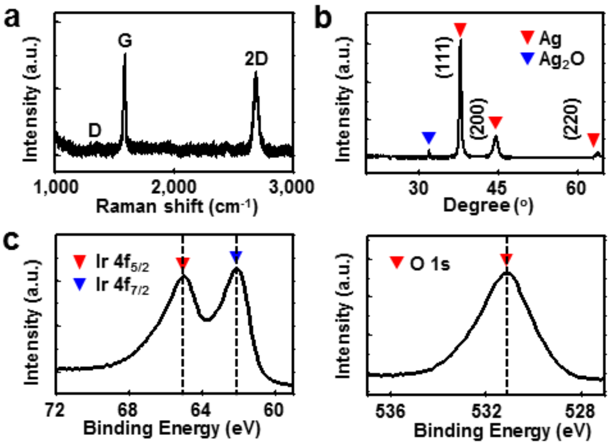

Supplementary Figure 5 | Material characterizations of graphene-hybrid. (a) Raman spectroscopy data of the synthesized graphene. **(b)** XRD (X-ray diffraction) data of Ag NWs. **(c)** XPS (X-ray photoelectron spectroscopy) data of the electroplated IrO_x film.

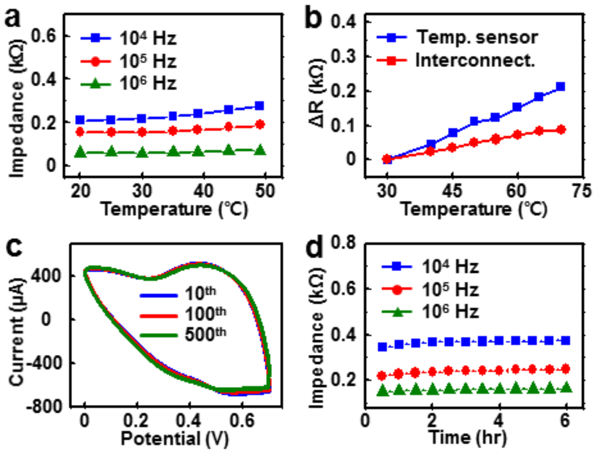

Supplementary Figure 6 | Electrical stability of graphene-hybrid under biofluidic environments. (a) Stable impedance of graphene-hybrid at different temperatures and frequencies of the alternating current. **(b)** Temperature dependent resistance changes of the temperature sensor and interconnection. Temperature sensors show higher resistance changes than the interconnection for temperature changes. **(c)** Stability of the electrodeposited IrO_x film during cyclic voltammetry measurements. **(d)** Stable impedance of the electroplated IrO_x film in the fetal bovine serum.

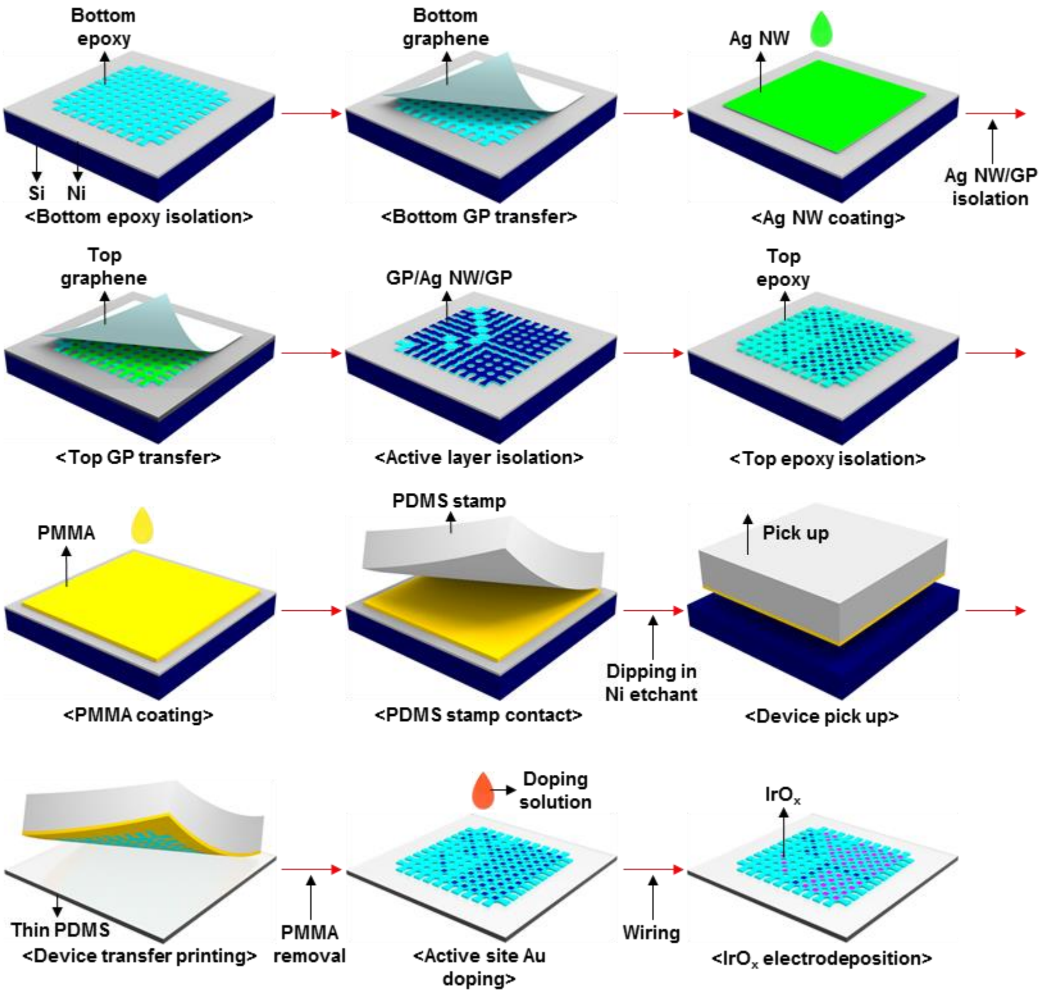

Supplementary Figure 7 | Device fabrication process. Schematic illustrations of the microfabrication process of transparent bio-electronic devices on a handle substrate.

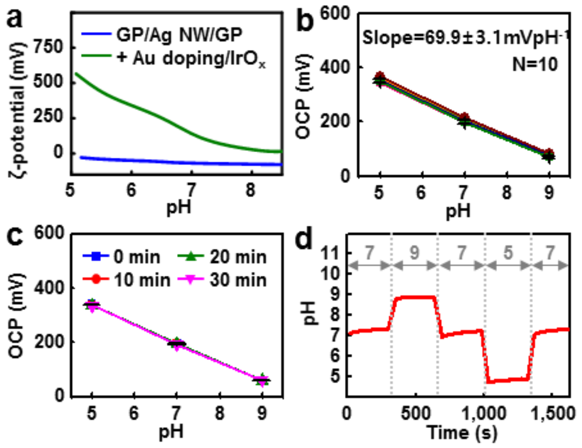

Supplementary Figure 8 | Characterization of the graphene-hybrid pH sensor. (a) Zeta potential changes of GP/Ag NW/GP and graphene-hybrid at different pHs. **(b)** Open circuit potential changes as a function of pH, measured by using 10 different pH sensors. **(c)** Open circuit potential changes as a function of pH in repeated uses with different time scales. **(d)** Characterization of pH sensors in standard buffer solutions whose pH changes 7→9 →7 →5 →7.

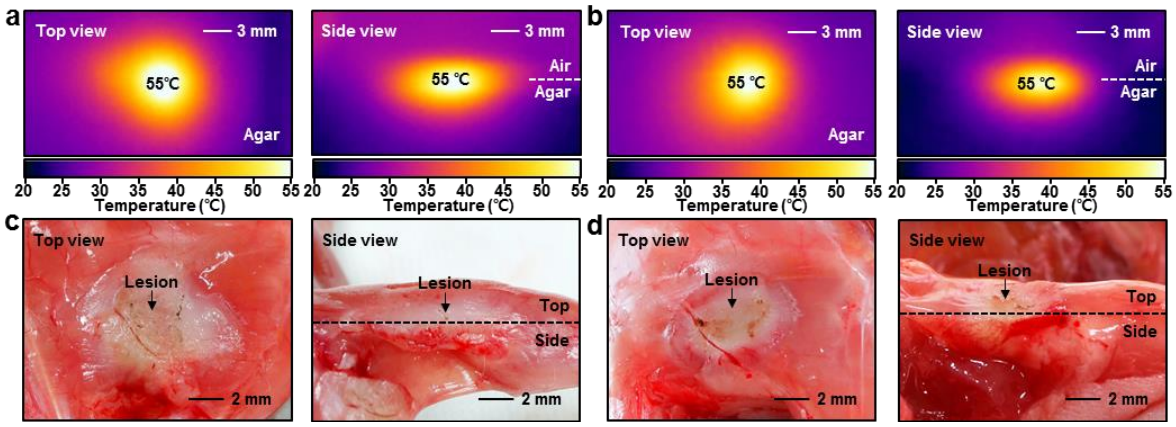

Supplementary Figure 9 | Comparison of the graphene-hybrid RF ablation electrode to conventional commercial RF ablation electrode. (a) IR images of agar during RF ablation using the graphene-hybrid based electrode (left: top view, right: side view). **(b)** IR images of agar during RF ablation using conventional commercial ablation electrode (left: top view, right: side view). **(c)** Optical camera images of the lesion size and depth on the mouse thigh muscle after RF ablation *in-vivo* using the graphene-hybrid ablation electrode (left: top view, right: side view). **(d)** Optical camera images of the lesion size and depth on the mouse thigh muscle after RF ablation *in-vivo* using conventional commercial ablation electrode (left: top view, right: side view).

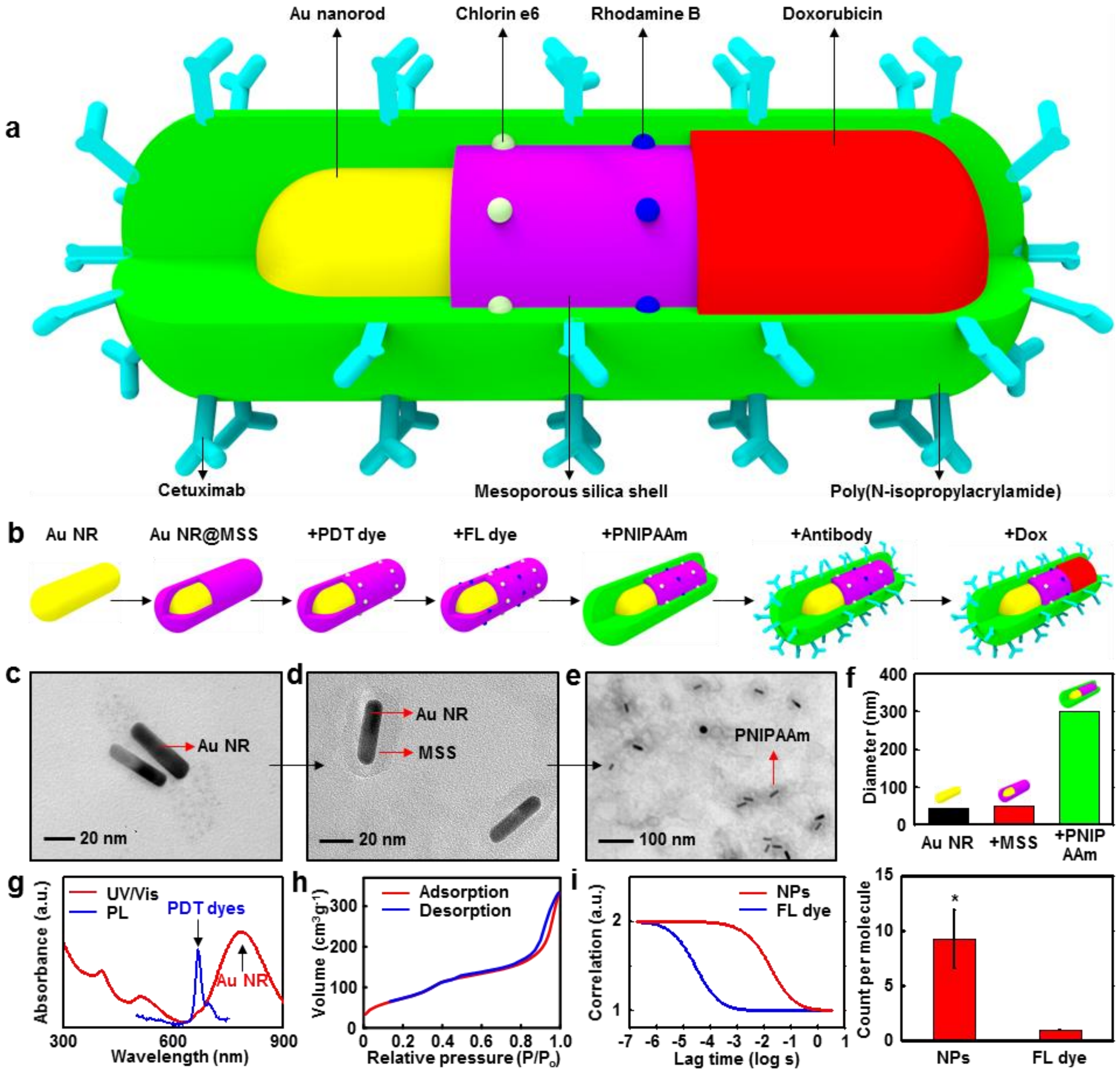

Supplementary Figure 10 | Characterization of multifunctional theranostic NPs. (a) Detailed schematic illustrations of theranostic NPs. **(b)** Schematic illustrations of the synthesis process of multifunctional theranostic NPs. **(c)** TEM image of Au NRs. **(d)** TEM image of the Au NR@MSS. **(e)** TEM image of the Au NR@MSS@PNIPAAm. **(f)** Hydrodynamic diameters measured by the dynamic light scattering at room temperature. **(g)** Ultraviolet-visible absorption and photoluminescence spectroscopy data of Au NR@MSS@PDT dye. Characteristic absorption peaks for Au NR (PTT) and PDT dyes are shown. **(h)** BET (Brunauer-Emmett-Teller) characterization result for the estimation of the surface area of Au NR@MSS. **(i)** Florescence correlation spectroscopy data of NPs and FL dyes. Conjugation of fluorescent dyes on NPs is shown by correlation curve (left) and normalized count per molecule (right) (*$p < 0.01$, Student's t-test).

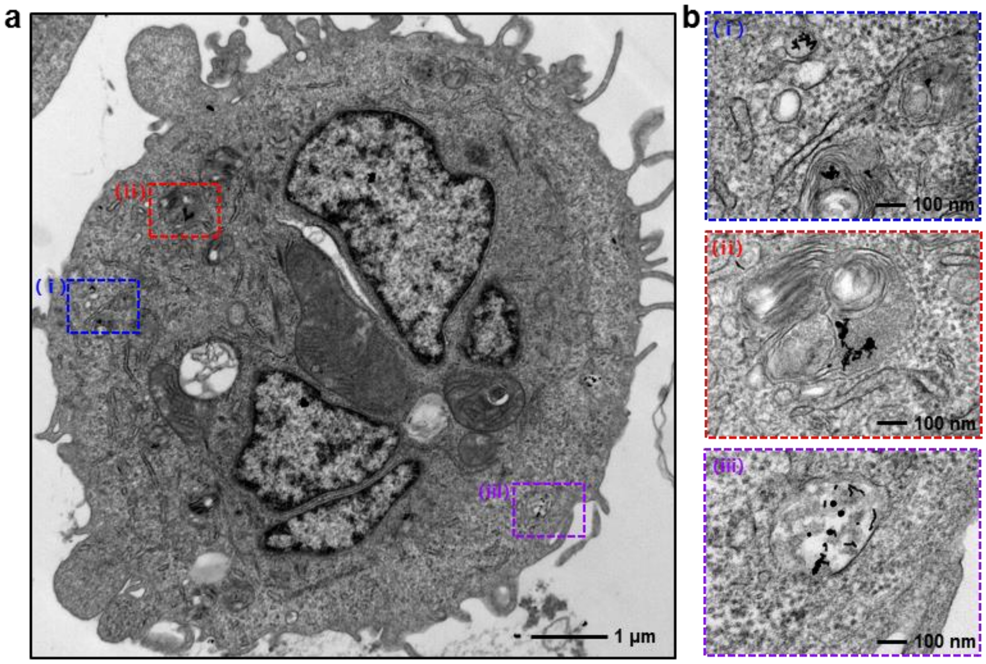

Supplementary Figure 11 | TEM observation of the cellular uptake of theranostic NPs. (a) Cell TEM image of theranostic NPs uptaken by a HT-29 cancer cell at the low magnification. **(b)** Cell TEM images of theranostic NPs uptaken by a HT-29 cancer cell at the high magnification.

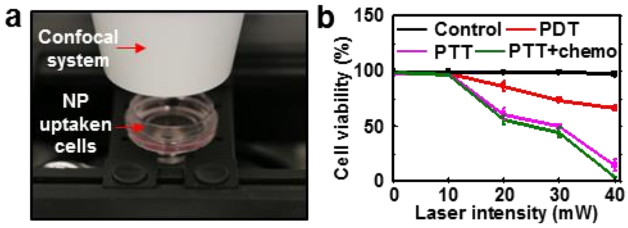

Supplementary Figure 12 | Optimization of photo-therapies. (a) Localized optical activation of theranostic NPs by using the laser irradiation of the confocal microscope. **(b)** Cell viability results of control- and NP-targeted- HT29 after various photo-therapies (PDT, PTT, PTT+chemo) with the modulation of the laser power.

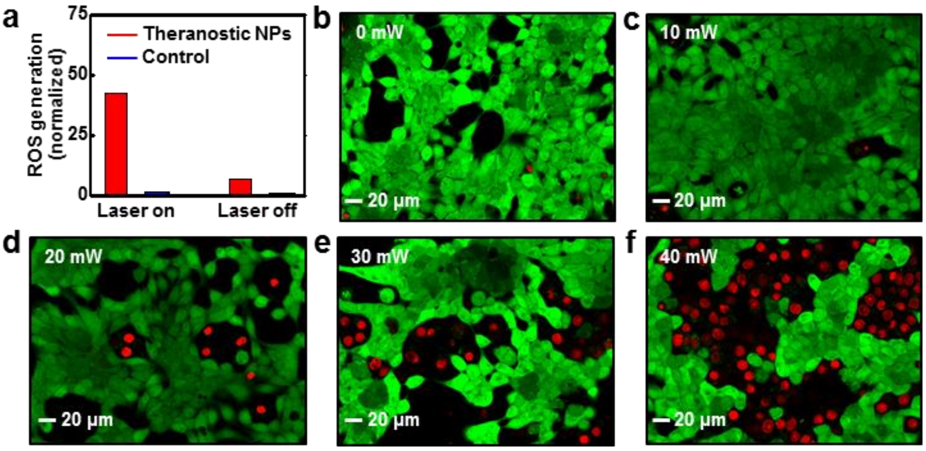

Supplementary Figure 13 | Optimization of photodynamic therapy. (a) Measurement of the ROS generation by theranostic NPs using singlet oxygen sensor green reagent before and after CW red laser irradiation. **(b-f)** Optimization of photodynamic therapy by modulating the power of pulsed red laser.

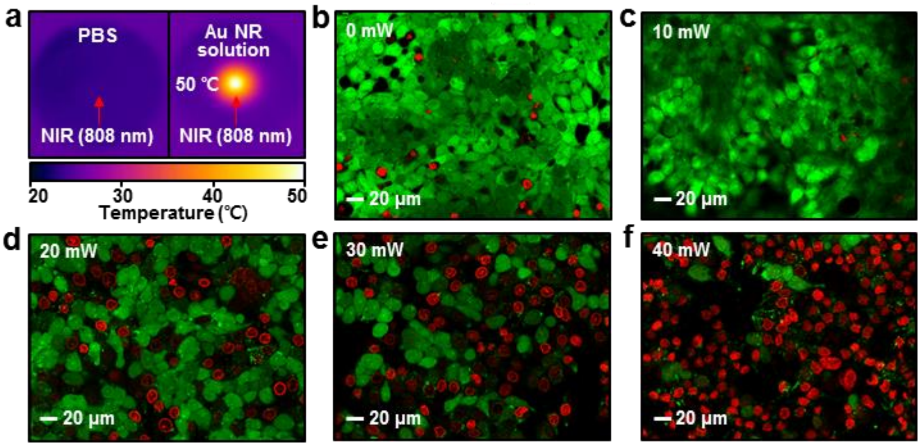

Supplementary Figure 14 | Optimization of photothermal therapy. (a) Localized photothermal activation of theranostic NPs with pulsed NIR laser irradiation. **(b-f)** Optimization of photothermal therapy by modulating the power of pulsed NIR laser.

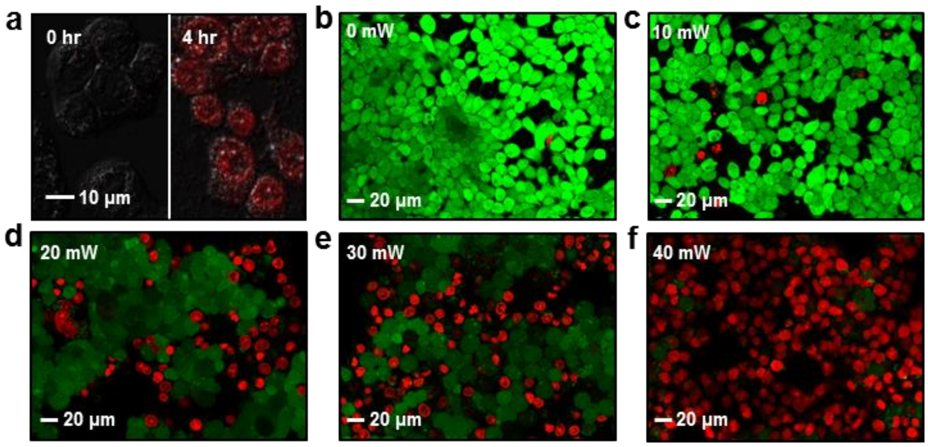

Supplementary Figure 15 | Optimization of chemotherapy. (a) Confocal microscope images of Dox release (left: 0 hour, right: 4 hours) from theranostic NPs treatment. Red fluorescence means Dox. **(b-f)** Optimization of the chemo-therapy by modulating the power of pulsed NIR laser.

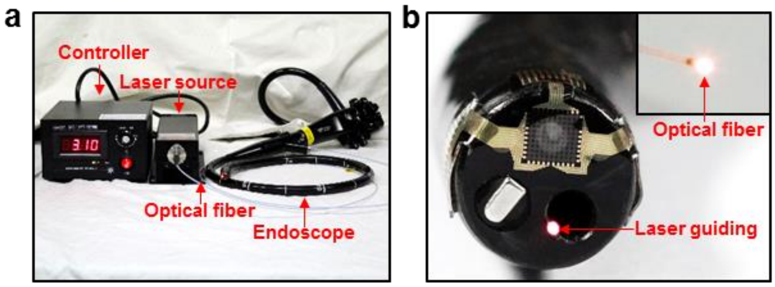

Supplementary Figure 16 | Laser guiding system through the instrumented surgical endoscope. (a) Image of the laser guiding system through the endoscope using the optical fiber. **(b)** Optical-fiber-guided laser irradiation through the endoscope. Inset shows the magnified view of the laser from the optical fiber tip.

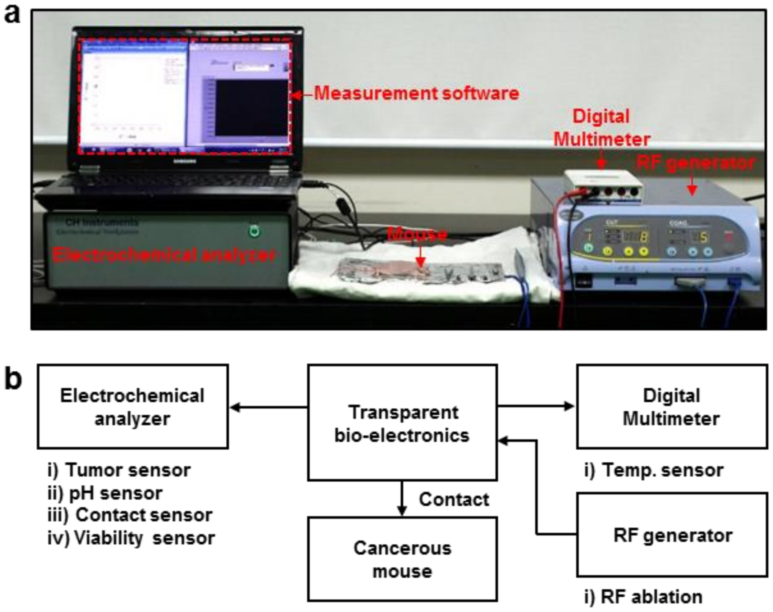

Supplementary Figure 17 | *In-vivo* experimental setup of the transparent bio-electronics. **(a)** Image of the *in-vivo* experimental setup of the transparent bio-electronic system including the electrochemical analyzer and the RF ablation therapy equipment. **(b)** Schematic diagram of the *in-vivo* experimental setup of the transparent bio-electronic system.

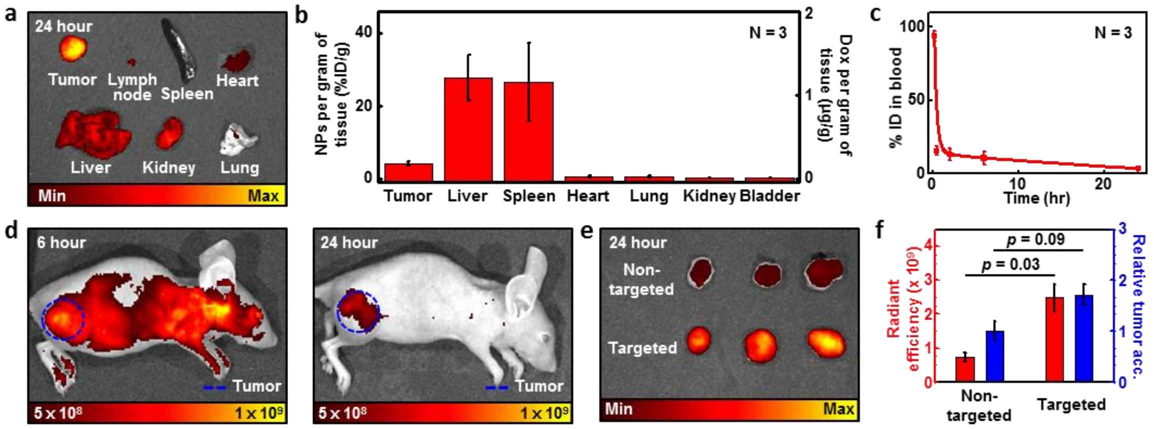

Supplementary Figure 18 | *In-vivo* analysis of the active targeting and biodistribution of theranostic NPs. (a) IVIS *ex-vivo* organ images after 6 hours from the IV injection of NPs. **(b)** ICP-MS analysis data that show the bio-distribution of NPs after 24 hours from the IV injection (left axis: biodistribution data of theranostic NPs in each organ based on the percentage of injected dose per gram tissue, right axis: biodistribution data of drug in each organ) (mouse number = 3). **(c)** Pharmacokinetic data for theranostic NPs in the mouse model ($t_{1/2}$ = 20 min) (mouse number = 3). **(d)** IVIS whole body images after intravenous injection of theranostic NPs (left: 6 hours, right: 24 hours). **(e)** IVIS *ex-vivo* tumor images of non-targeted and targeted NPs after 24 hours from the intravenous injection (mouse number is three for each experimental group). **(f)** ROI (region of interest) values in IVIS *ex-vivo* tumor images of Supplementary Fig. 18e (p = 0.03, Student's t-test) and corresponding normalized ICP-MS analysis data by the amount of theranostic NPs in non-targeted tumors (p = 0.09, Student's t-test) (mouse number is three for each experimental group).

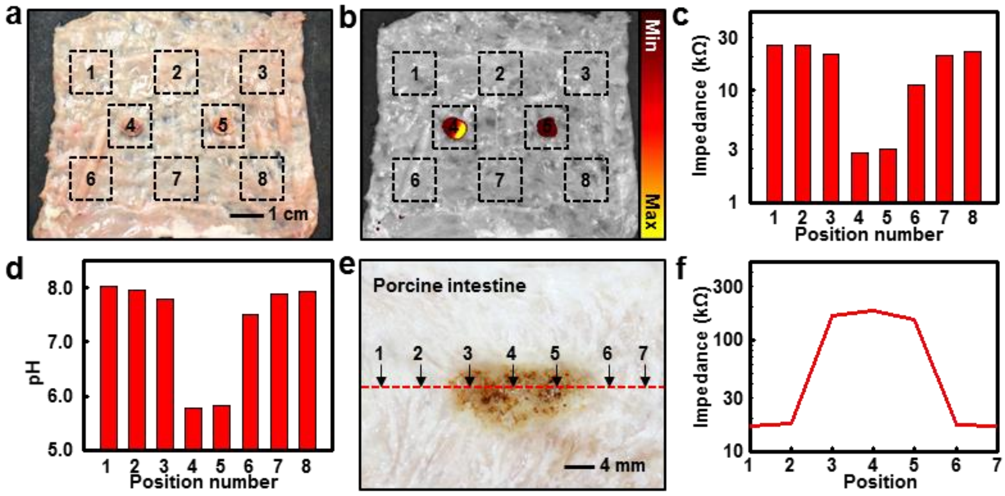

Supplementary Figure 19 | *Ex-vivo* tumor mapping through the theranostic NPs and the transparent bio-electronics. (a) An optical camera image of nanoparticle-targeted tumors attached to porcine intestine. The tumor is resected from tumor tissues grown on subdermal tissues of mouse. **(b)** IVIS fluorescence image corresponding to Supplementary Fig. 19a. **(c)** Impedance mapping of tumors attached porcine intestine by using the tumor sensor (operation frequency: 10^4 Hz). The sensing positions are shown in Supplementary Fig. 19a. **(d)** pH mapping of tumors attached porcine intestine by using the pH sensor. The sensing positions are shown in Supplementary Fig. 19a. **(e)** An optical camera image of porcine intestine after RF ablation. **(f)** Viability mapping of porcine intestine after RF ablation by using the viability sensor. The sensing positions are shown in Supplementary Fig. 19e (operation frequency: 10^4 Hz).

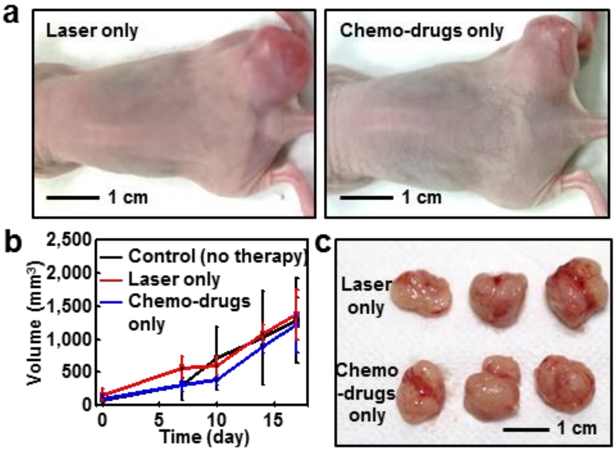

Supplementary Figure 20 | *In-vivo* tumor treatments without theranostic NPs. (a) Images of the mouse model with HT-29 tumors (left: after 808 nm laser irradiation only, right: after chemo-drugs (Dox) therapy only). Two treated groups without theranostic NPs show the tumor growth. **(b)** Summary of tumor volume changes of Supplementary Fig. 20a with the control group (mouse number is three for each experimental group). **(c)** Extracted tumor images after treatments (up: 808 nm laser irradiation only, bottom: chemo-drugs therapy

only).

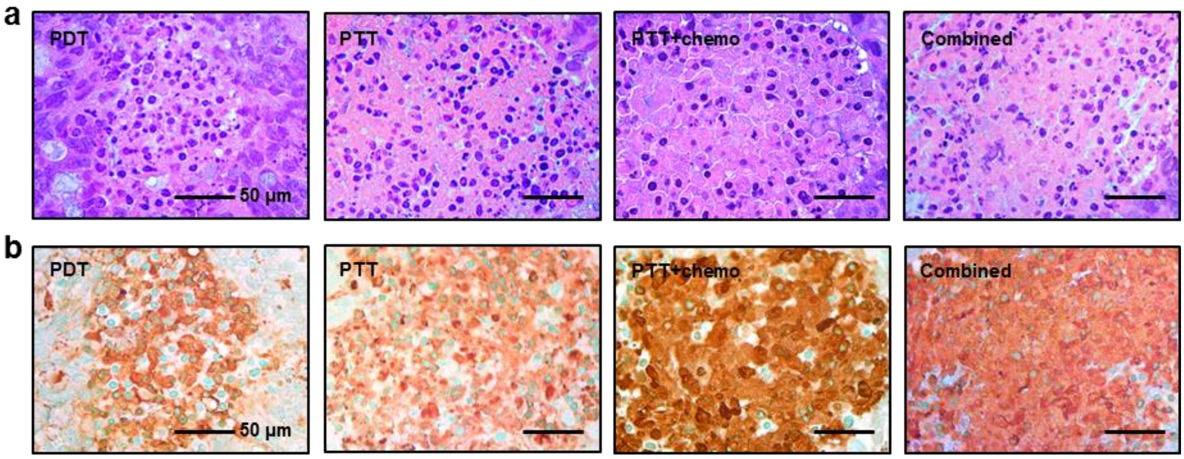

Supplementary Figure 21 | H&E and TUNEL assay after photo-therapies using theranostic NPs *in-vivo*. (a) Hematoxylin and eosin (H&E) staining images of HT-29 tumor tissues after PDT, PTT, PTT+chemo and combined therapy. **(b)** TUNEL assay images of HT-29 tumor tissues after PDT, PTT, PTT+chemo and combined therapy.

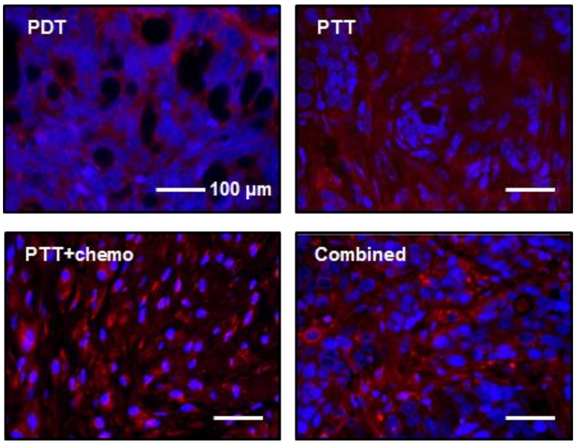

Supplementary Figure 22 | DAPI and cleaved caspase-3 staining of HT-29 tissues after PDT, PTT, PTT+chemo, and combined therapy.

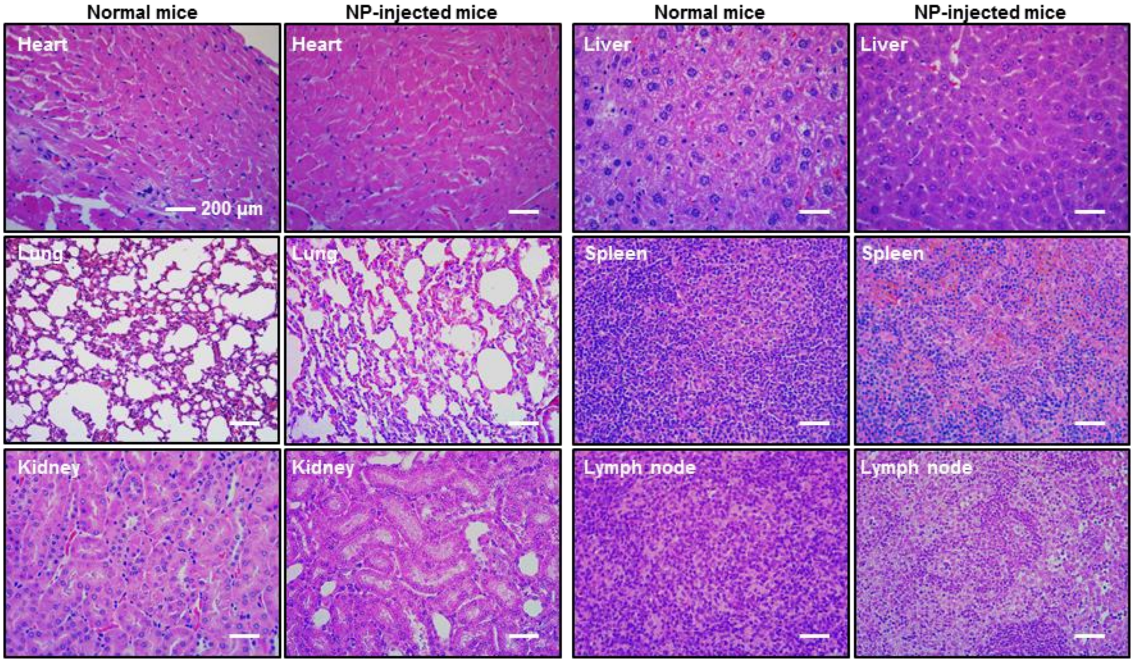

Supplementary Figure 23 | *In-vivo* nanoparticle toxicity. Hematoxylin and eosin (H&E) stained tissue images from various organs of normal and NP-injected mouse. Theranostic NPs cause no inflammations on individual organs, which means that NPs have no toxicity.

Supplementary Note 1

Mechanical and thermal stability of transparent bio-electronics. Indium tin oxide (ITO) is typically used in transparent electronics, but it is vulnerable to mechanical failures during installation, disassembly, and use (Supplementary Fig. 2a,b). In contrast, transparent bio-electronics offer better material properties during repetitive contact, rubbing, bending and twisting deformations (Supplementary Fig. 2d-f). The ultrathin format of transparent bio-electronics further helps to achieve a high degree of mechanical deformability. Thermal stability (Supplementary Fig. 2c) is characterized through electrical performance test before and after the sterilization (hot steam sterilization in an autoclave, 120 °C, 200 kPa, 15 min).

Supplementary Note 2

Electrodeposition of IrO_x films on graphene. Hydrous IrO_x film electrodeposited on the graphene has low interfacial impedance, minimal volume expansion, and high pH sensitivity. IrO_x film is, therefore, suitable for detection of small electrical signals, ablation energy delivery, measurement of pH changes, and electrical charge injections into tissues[1,2]. Au-doping of a GP/Ag NW/GP structure reduces the contact angle and enhances surface wetting in aqueous solutions containing IrO_x precursors (Supplementary Fig. 4a,b). This results in uniform coating of IrO_x, as shown in SEM images of Supplementary Fig. 4c–f. More importantly, the low impedance property created by IrO_x deposition improves signal to noise ratio and charge injection efficiencies of transparent bio-electronics.

Supplementary Note 3

Material characterization of transparent bio-electronics. The graphene component of the hybrid is characterized by Raman spectroscopy (Supplementary Fig. 5a). It is composed of 2-3 graphene layers, based on the relative peak intensity of the 2D and G band ($I_{2D/G}$=1.01). Moreover, the defect-related D band peak is strongly suppressed, indicating the synthesis of high-quality graphenes. Ag NWs are analyzed by X-ray diffraction (XRD; Supplementary Fig. 5b). The detection of a peak of Ag_2O ($\theta=32^o$) indicates a small amount of silver oxide formed on the surface during thermal annealing. Further oxidation is minimized by passivation with the top graphene[3]. The electrodeposited IrO_x film is characterized by X-ray photoelectron spectroscopy (XPS; Supplementary Fig. 5c)[4], with the Ir $4f_{7/2}$ and Ir $4f_{5/2}$ peak energies of 62.1 and 65.0 eV, indicating bonds between Ir and O atoms. In comparison with characteristic XPS peaks of metallic Ir (61.1 and 64.1 eV) and an IrO_2 standard (62.7 and 65.7 eV), the electrodeposited IrO_x exists in highly oxidized forms.

Supplementary Note 4

Impedance measurement of tumor and normal tissues. Cancerous tissue exhibits significantly lower impedance compared to healthy tissue. This difference in impedance has been used to detect tumor growths. Bio-impedance recordings are highly dependent on the size, spacing, and density, which can alter electrical signals and charge storage in tissues. The specific frequency range is also important for tissue discrimination based on bio-impedance analysis. Impedance-based tumor detection has been used in the diagnosis of breast, esophageal, prostate and brain cancers[5-7] by discriminating the impedance of normal tissues from that of tumors. Previous studies compare impedances across cancerous tissues and corresponding normal tissues in the range between 10 kHz and 1 MHz (*e.g.* breast tumor tissues are compared to nearby normal breast tissues). The endoscopic sensing study presented in this work demonstrates *in-vivo* analysis of sub-cutaneous colon cancer (HT-29)

model in a BALB/c-nude mouse. Therefore, the biosensor measures impedance differences between cancer tissues and normal dermis tissues. In both *ex-vivo* (Fig. 3d middle) and *in-vivo* (Fig. 5d) experiments, colon cancer (HT-29) tissue exhibits lower impedances than normal tissues. The Student's t-test for the impedance sensor is carried out *ex-vivo* and *in-vivo*. The p value is statistically significant ($p < 0.001$) for each experiment, which means that the impedance sensor has the high sensitivity and specificity for the tumor detection. The specificity of the impedance sensor can be complemented by actively targeted nanoparticle based fluorescence imaging (Fig. 5a).

Supplementary Note 5

pH measurement of tumor and normal tissues. Extracellular pH level of cancerous tissues is lower than that of normal tissues due to increased lactic acid production and reduced interstitial fluid buffering. Previous studies have shown direct imaging of tumor acidity[8]. This acidity mapping result can be used for the tumor diagnosis. Our pH sensor is used to measure pH around tumors as a way to differentiate tumor tissues from normal tissues. Previous flexible pH sensors have used three-electrode method that is for the solution phase pH measurement. We develop a two-electrode-based pH sensor. The pH sensor consists of an IrO_x electrodeposited graphene-hybrid for working electrode and an Au-doped GP/Ag NW/GP electrode for the counter electrode (Fig. 3d top). This is due to the pH sensitive property of electrodeposited IrO_x (Supplementary Fig. 9a). The pH sensor is reproducible for different pH sensors, during multiple use and pH variation of buffer solution (Supplementary Fig. 8b-d). The sensors are calibrated (Fig. 3d bottom) and pH values of cancerous and normal tissues are measured *in-vivo* (Fig. 5e). The Student's t-test for the pH sensor is carried out *ex-vivo* and *in-vivo*. The p value is statistically significant ($p < 0.001$) for each experiment, which means that the pH sensor has the high sensitivity and specificity for the tumor detection. The specificity of the pH sensor can be complemented by actively targeted nanoparticle based fluorescence imaging (Fig. 5a).

Supplementary Note 6

Synthesis of theranostic NPs. The synthesis of NPs is described in Supplementary Fig. 10a,b and the TEM images of each step are provided in Supplementary Fig. 10c–e. The real and hydrodynamic size of NPs is shown in the TEM images and in the dynamic light scattering measurement data at room temperature (Supplementary Fig. 10f). Au NRs are characterized by the absorption peaks at 540 nm (transversal) and 800 nm (longitudinal) in the UV-visible spectrometry measurement (Supplementary Fig. 10g). The longer wavelength of longitudinal absorption (relative to transverse) is due to the aspect ratio (~4) of the nanorod (8×30 nm). Since 800 nm wavelength is the optical window (the wavelength region of the high penetration depth) of our body, a NIR laser can hyperthermally activate Au NRs. The surface area of the synthesized MSS on Au NRs is sufficiently large (BET data in Supplementary Fig. 10h) to load FL dyes, PDT dyes and chemo-drugs (Dox). The amount of drugs loaded and PDT dyes (Chlorine 6) conjugated on NPs are 0.86 g Dox per g Au and 0.06 g PDT dye per g Au, respectively, which are measured by using UV-vis spectrophotometer and ICP-AES. Several different concentrations of NPs for the uptake into HT-29 cancer cells are used for the ICP-MS analysis to identify the amount of NPs (Au mg per cell), which is necessary to identify for effective phototherapy. The results show that more than 250 NPs per one HT-29 cancer cell are needed to treat the cancer cells. Florescence correlation spectroscopy (FCS) data confirm that FL dyes are well conjugated on the surface of MSS (10

FL dyes per NP) (Supplementary Fig. 10i). PNIPAAm is thermo-sensitive to volume change, with a critical temperature near the body temperature (36.5 °C), Dox can be inadvertently released into other organs at body temperature. Therefore, the critical temperature of the volume change is modified by making block-copolymers from N-isopropylacrylamide and acrylic acid monomers[9]. This modification of PNIPAAm increases the critical volume change temperature from 35 °C to >45 °C (Fig. 4g middle). The outer polymer layer is functionalized with a hetero-functionalized PEG (acrylate-PEG-NHS) to allow the conjugation with antibodies for the active targeting.

Supplementary Note 7

In-vivo **biodistribution and pharmacokinetic analysis.** Fluorescence-based tumor detection is widely used in nano-science[10]. The fluorescence-based tumor mapping by using 5-aminolevulinic acid is being used in the brain tumor surgery[11]. After the intravenous (IV) injection, NPs circulate in the blood stream and accumulate at targeted tumor sites due to antigen-antibody reactions (active targeting) as well as enhanced permeability and retention (EPR) effect (passive targeting). The targeted (antibody-conjugated) nanoparticles are delivered to the tumor more efficiently in comparison with non-targeted ones (Supplementary Fig. 18e,f). IVIS images show that the injected NPs are targeted to tumor effectively after 6 hours of injection (Fig. 5a) and mainly remain in tumor after 24 hours of injection (Supplementary Fig. 18d). NPs also tend to gather in the lymph nodes, spleen and liver. However NPs rarely accumulate in kidneys and lungs, due to their large size (Supplementary Fig. 18a,b). In order to determine the quantity of NPs still circulating in blood, the Au content in mouse blood is measured by an inductively coupled plasma-mass spectrometer (ICP-MS). Approximately half of injected NPs remain circulating in the bloodstream after 20 min ($t_{1/2}$=20 min) (Supplementary Fig. 18c).

Supplementary References

1) Meyer, R. D., Cogan, S. F., Nguyen, T. H. & Rauh, R. D. Electrodeposited iridium oxide for neural stimulation and recording electrodes. *IEEE Trans. Neural. Syst. Rehabil. Eng.* **9**, 2-11 (2001).
2) Weiland, J. D. & Anderson, D. J. Chronic neural stimulation with thin-film, iridium oxide electrodes. *IEEE Trans. Biomed. Eng.* **47**, 911-918 (2000).
3) Chen, S. *et al.* Oxidation resistance of graphene-coated Cu and Cu/Ni Alloy. *ACS Nano* **5**, 1321-1327 (2011).
4) Sawy, E. N. E. & Birss, V. I. Nano-porous iridium and iridium oxide thin films formed by high efficiency electrodeposition. *J. Mater. Chem.* **19**, 8244-8252 (2009).
5) Kerner, T. E. *et al*. Electrical impedance spectroscopy of the breast: clinical imaging results in 26 Subjects. *IEEE. Trans. Med. Imag.* **21**, 638-645 (2002).
6) Halter, R. J. *et al*. Electrical impedance spectroscopy of the human prostate. *IEEE Trans. Biomed. Eng.* **54**, 1321-1327 (2007).
7) Jahnke, H.-G. *et al*. Impedance spectroscopy-an outstanding method for label-free and real-time discrimination between brain and tumor tissue in vivo *Biosens. Bioelectron*. **46**, 8-14 (2013).
8) Urano Y. *et al*. Selective molecular imaging of viable cancer cells with pH-activatable fluorescence probes. *Nat. Med.* **15**, 104-109 (2009).
9) Yoo, M. K. Yoo *et al.* Effect of polymer complex formation on the cloud-point of poly(N-

isopropyl acrylamide) (PNIPAAm) in the poly(NIPAAm-co-acrylic acid): polyelectrolyte complex between poly(acrylic acid) and poly(allylamine). *Polymer* **38**, 2759-2765 (1997).
10) Vahrmeijer, A. L. *et al.* Image-guided cancer surgery using near-infrared fluorescence. *Nat. Med.* **10**, 507-518 (2013).
11) Stummer, W. *et al.* Fluorescence-guided surgery with 5-aminolevulinic acid for resection of malignant glioma: a randomised controlled multicentre phase III trial. *Lancet Oncol.* **7**, 392-401 (2006).
